# Fluorescent *salen*-type Zn(II) Complexes As Probes for Detecting Hydrogen Sulfide and Its Anion: Bioimaging Applications

**DOI:** 10.1021/acs.inorgchem.0c02499

**Published:** 2020-10-13

**Authors:** Maria Strianese, Daniela Guarnieri, Marina Lamberti, Alessandro Landi, Andrea Peluso, Claudio Pellecchia

**Affiliations:** Dipartimento di Chimica e Biologia “Adolfo Zambelli”, Università degli Studi di Salerno, Via Giovanni Paolo II, 132, 84084 Fisciano, Salerno, Italy

## Abstract

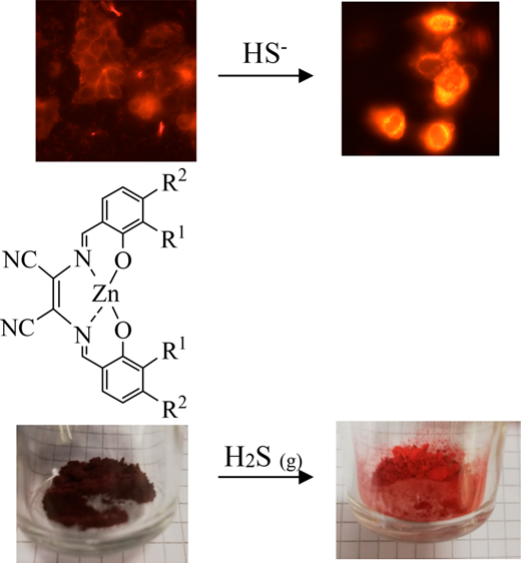

In this work, we investigate the mode of interaction of a family of fluorescent zinc complexes with HS^–^ and H_2_S. Different experiments, performed by diverse spectroscopic techniques, provide evidence that HS^–^ binds the zinc center of all the complexes under investigation. Treatment with neutral H_2_S exhibits a markedly different reactivity which indicates selectivity for HS^–^ over H_2_S of the systems under investigation. Striking color changes, visible to the naked eye, occur when treating the systems with HS^–^ or by an H_2_S flow. Accordingly, also the fluorescence is modulated by the presence of HS^–^, with the possible formation of multiple adducts. The results highlight the potential of the devised systems to be implemented as HS^–^/H_2_S colorimetric and fluorescent sensors. Bioimaging experiments indicate the potential of using this class of compounds as probes for the detection of H_2_S in living cells.

## Abstract 2

In this work, we studied the reactivity of HS^−^ with a family of fluorescent zinc complexes via a variety of spectroscopic techniques. Interaction with HS^−^ results in its coordination to the Zn center for all the complexes under investigation. Treatment with neutral H_2_S exhibits a markedly different reactivity which indicates selectivity for HS^−^ over H_2_S. Striking color changes, visible to the naked eye, occurred when treating the systems with HS^−^ or by an H_2_S flow.

## Introduction

Over the past two decades, hydrogen sulfide (H_2_S) has gained increasing attention as a biological molecule which mediates important functions within the human body through its action on bioinorganic targets, joining NO and CO in the family of gasotransmitters. By now, the biological reactivity of NO and CO has been widely clarified thanks to the numerous papers focusing on their coordination chemistry to bioinspired metal complexes.^1−4^ Differently, H_2_S reactivity is still in a premature stage mainly owing to the fact that H_2_S is a weak acid, which in aqueous solution equilibrates with its anions HS^–^ and S^2–^ (at physiological pH (7.4), 28% of the total hydrogen sulfide exists as H_2_S, 72% is in the form of HS^–^, whereas S^2–^ is negligible)^5^ thus complicating the studies on H_2_S reactivity in biological media and the clarification of the specific chemistry associated with the specific protonation state.^6^ In particular, finding a way to differentiate the reactivity of H_2_S from that of HS^–^ is a challenging task. Stable H_2_S/HS^–^ adducts of biomimetic metal complexes are still not numerous because of the propensity of metal sulfides to precipitate in addition to the redox reactivity of sulfides.^6−9^

Drawing upon these considerations, some time ago, we and others focused our efforts on the study of the coordination of H_2_S/HS^–^ to transition metals.^10−14^ In particular, we explored both the reactivity of properly tailored molecular complexes and that of natural metalloproteins.^15−23^ More recently, we focused on zinc porphyrins and on zinc tetradentate Schiff-based complexes which share many structural features (i.e., both tetradentate and planar) targeting these systems as viable scaffolds for isolating and characterizing hydrosulfido species.^24−27^ Indeed, among the wide number of d^10^ metal complexes, zinc(II) complexes with nitrogen-containing ligands are excellent candidates for the development of luminescent materials. In this context, blue-emitting zinc complexes (with a saturated chain between the two bridging nitrogens) attracted a lot of interest over the years.^28−32^ By substituting the bridge between the coordinating nitrogens with a conjugated spacer like, for instance, the maleonitrile unit, zinc complexes exhibiting red fluorescence and aggregation-enhanced emission have been reported in the past.^33−42^

**Scheme 1:**
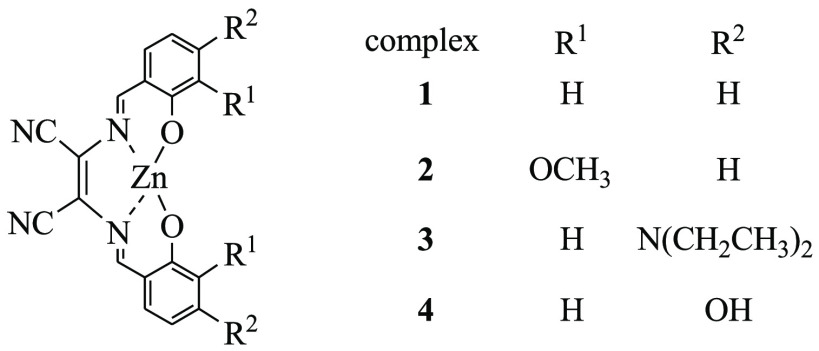


Herein, we decided to test the potential of a family of diaminomaleonitrile (DAMN)-based salen-type zinc complexes (see Scheme 1)^33,38,43^ to bind HS^–^ at the zinc center and their possible application as HS^–^ fluorescent sensors via a coordinative based approach.

The modifications on the ligand structure (see Scheme 1) were realized to study the possible effects on the zinc hydrosulfido stabilization. We also wanted to explore whether these different groups on the ligand structure would tune somehow the fluorescence properties of the related complexes as HS^–^ sensors.

## Results and Discussion

Complexes **1**–**4** were synthesized via template synthesis from DAMN and the proper substituted salicylaldehyde by following literature procedures.^33,38,43^ Characterization of the complexes was achieved by high-resolution MALDI Fourier transform ion cyclotron resonance mass spectrometry (HR MALDI-FT-ICR, Figures S1–S6) and ^1^H NMR analysis (Figures S7–S11).

### HS^–^ Response of Complexes **1**–**4** Studied via ^1^H NMR Spectroscopy

**Figure 1:**
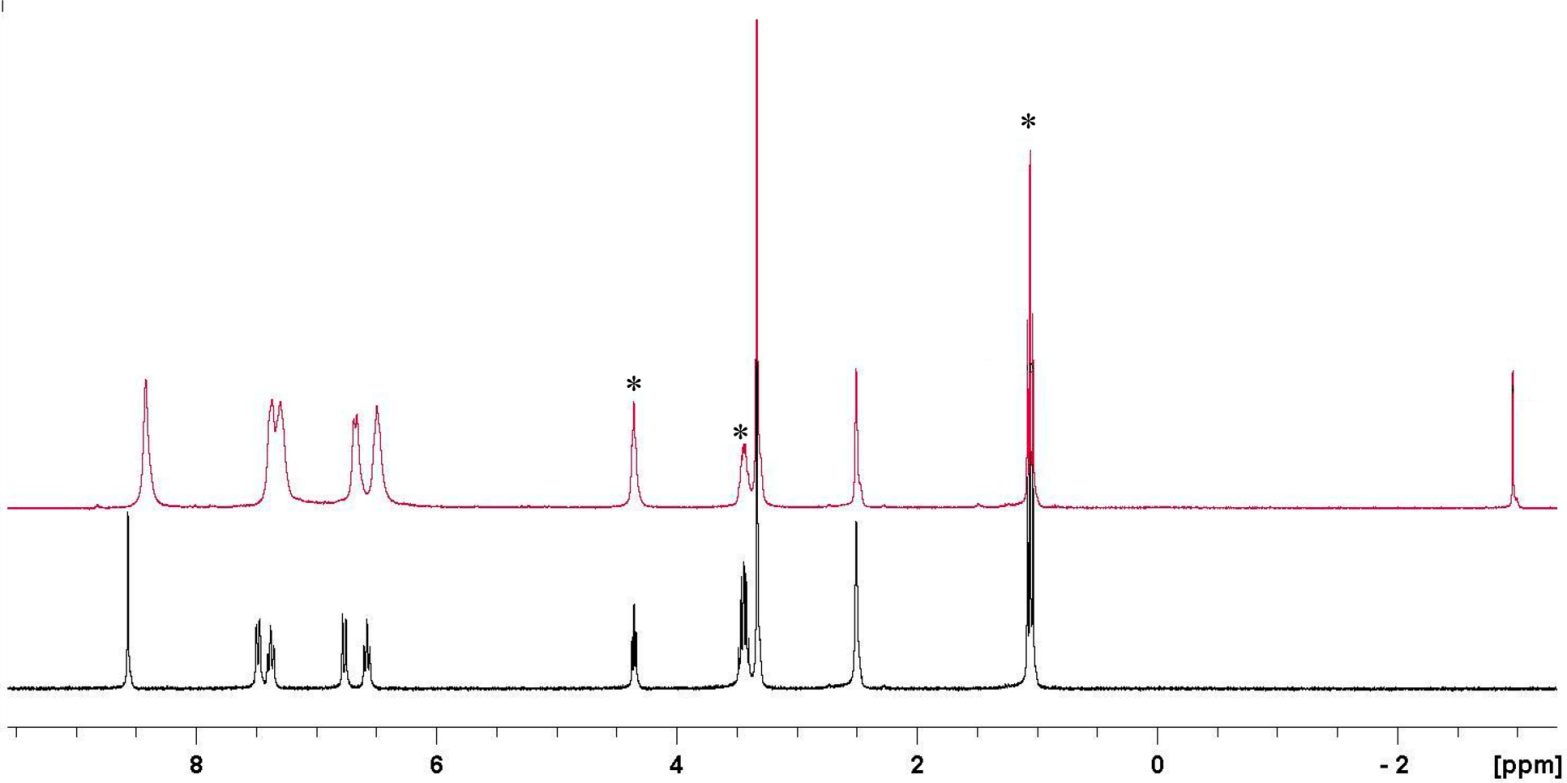
^1^H NMR spectra of complex **1** in DMSO-*d*_6_ (lower trace) and after the addition of an excess of HS^–^(upper trace). [complex **1**] = 5 × 10^–2^ M; [NaSH] = 0.25 M. Peaks denoted with * correspond to ethanol used for the synthesis.

First, the potential of HS^–^ binding to the zinc centers was investigated via NMR. The addition of NaSH to a DMSO-*d*_6_ solution of complex **1** resulted in a shift of the proton resonances (Figure 1) and, most remarkably, in the appearance of a high field resonance at δ −2.95 ppm, ascribable to the SH group bound to the zinc center, in agreement with the spectra of zinc hydrogenosulfido complexes reported in the literature.^9,12,13,26,27^

When performing the same NMR experiment with the other complexes under investigation, very similar behaviors were observed (Figures S12–S15). To investigate whether the OH groups in complex **4** are involved in hydrogen-bonding interactions with the Zn–SH moiety, as we had previously observed in the framework of our studies,^25,26^ we performed a NOESY NMR experiment which revealed a NOE contact between the S***H*** and the O***H*** signals (Figure S16), supporting their involvement in hydrogen-bonding interactions with the Zn–SH moiety. Stabilization of a Zn(II) hydrosulfide complex by hydrogen-bond assistance of the ligand was previously reported by Pluth and co-workers.^7^

### HS^–^ Response of Complexes **1**–**4** Studied via UV–vis and Fluorescence Spectroscopy

**Figure 2:**
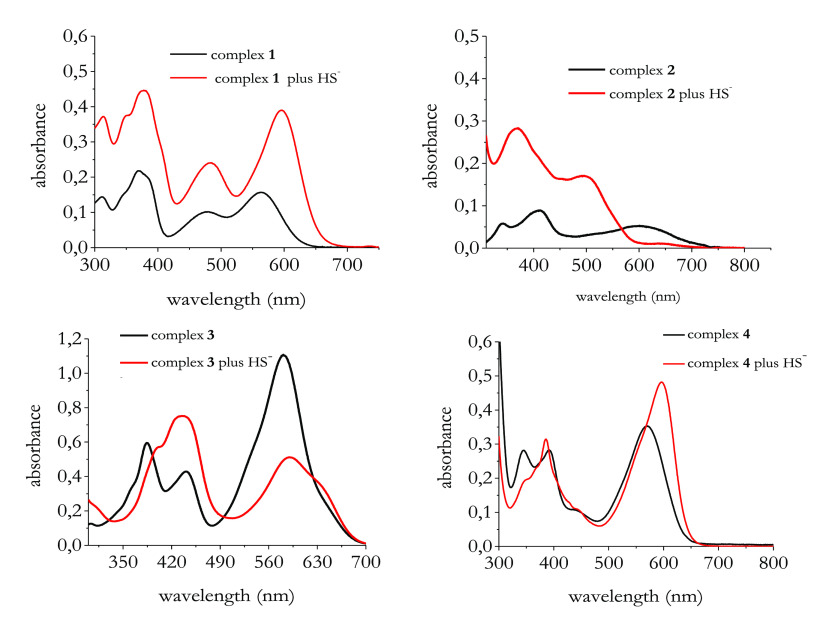
Electronic absorption spectra of complexes **1**–**4** with and without the addition of 50 μM of NaSH. Spectra recorded in DMSO. [Complexes] = 10 μM.

To test the optical properties of the complexes under investigation and their potential to act as HS^–^ fluorescence-based sensors, we started a study via UV–vis and fluorescence spectroscopy in DMSO. Figure 2 displays the absorption spectra of complexes **1**–**4** before and after interaction with HS^–^.

All the complexes under investigation show absorption bands both in the UV region (*ca*. 300–400 nm) and in the visible region (*ca*. 500–650 nm). In particular, the longer wavelength band can be attributed to intramolecular metal–ligand d → π* charge transfer transitions (MLCT), as for related systems.^33^ In the presence of HS^–^, visible changes of the initial absorption spectra of all the complexes under investigation occurred, thus confirming the formation of new species.

Next, we studied the fluorescence response of complexes **1**–**4** before and after HS^–^ addition.

**Figure 3:**
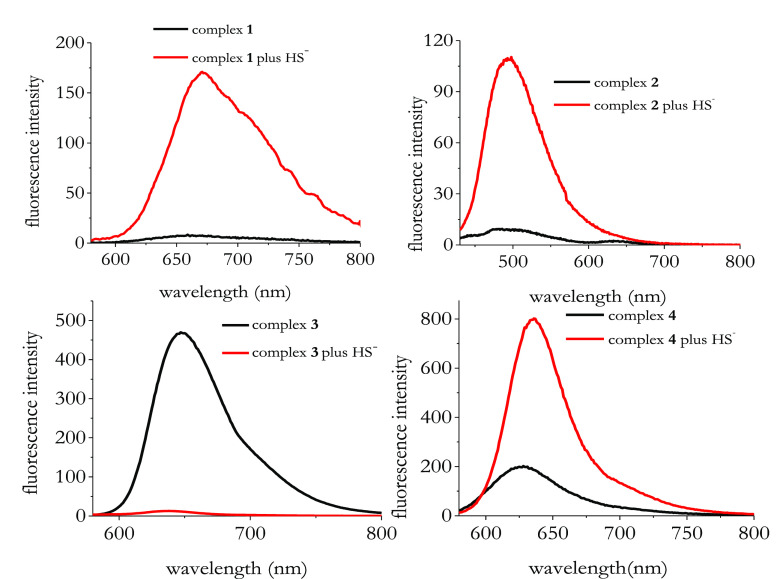
Emission spectra of complexes **1**–**4** before and after the addition of 5 equiv of NaSH. [Complexes **1**–**4**] = 1 × 10^–5^ M; [NaSH] = 5 × 10^–5^ M. All spectra were measured in DMSO with λ_exc_ = 560 nm for complex **1**; λ_exc_ = 414 nm for complex **2**; λ_exc_ = 442 nm for complex **3**; λ_exc_ = 570 nm for complex **4**.

As shown in Figure 3, complex **3** is the most fluorescent species: the higher fluorescence of complex **3** may be explained by the intramolecular charge transfer (ICT) effect, which is also known as the “push–pull” effect and was already reported in the case of salen ligands functionalized by an electron-donor (D, amine)/electron acceptor (A, cyanine) pair.^37,44^ Complex **3** displays the highest fluorescence quantum yield (Φ_F_) with respect to the other complexes under investigation (see the Experimental Section).

The addition of HS^–^ resulted in a significant fluorescence switching for all the complexes under investigation. In particular, complexes **1**, **2**, and **4** harnessed a sizable fluorescent enhancement, whereas complex **3** underwent a quenching of the initial fluorescence intensity.

In order to exclude the idea that the observed changes in the fluorescence spectra upon HS^–^ addition were simply due to pH variations or to some acid–base chemistry, we added a strong base (NaOH) to the DMSO solution of complex **4**. As shown in Figure S17, a fluorescence response different than that in the presence of NaSH (see Figure 3) was observed.

**Figure 4:**
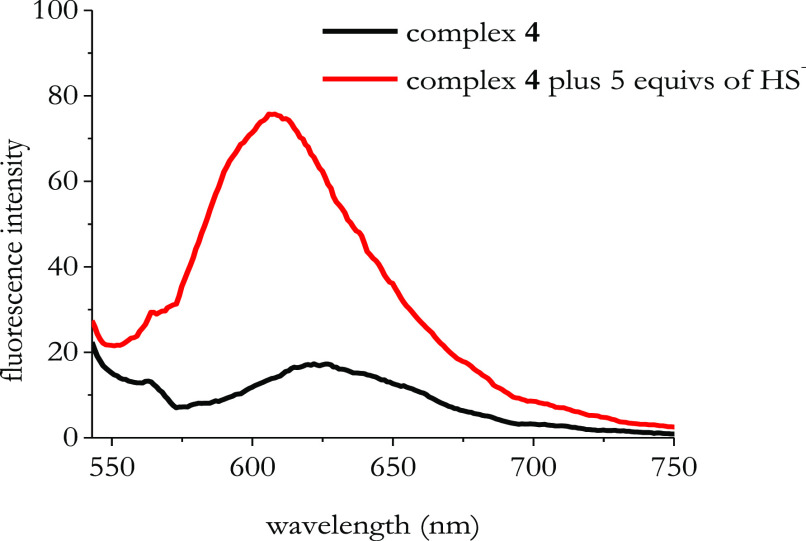
Emission spectra of complex **4** before and after the addition of 5 equiv of NaSH. [Complex **4**] = 1 × 10^–5^ M; [NaSH] = 5 × 10^–5^ M. Spectra were measured in MQ water with λ_exc_ = 530 nm.

In the course of our experiments, we found out that complex **4** is soluble in MQ water solution, which is a favorable condition for practical measurements in biological media. Figure 4 displays the fluorescence response of complex **4** in MQ water solution.

**Figure 5:**
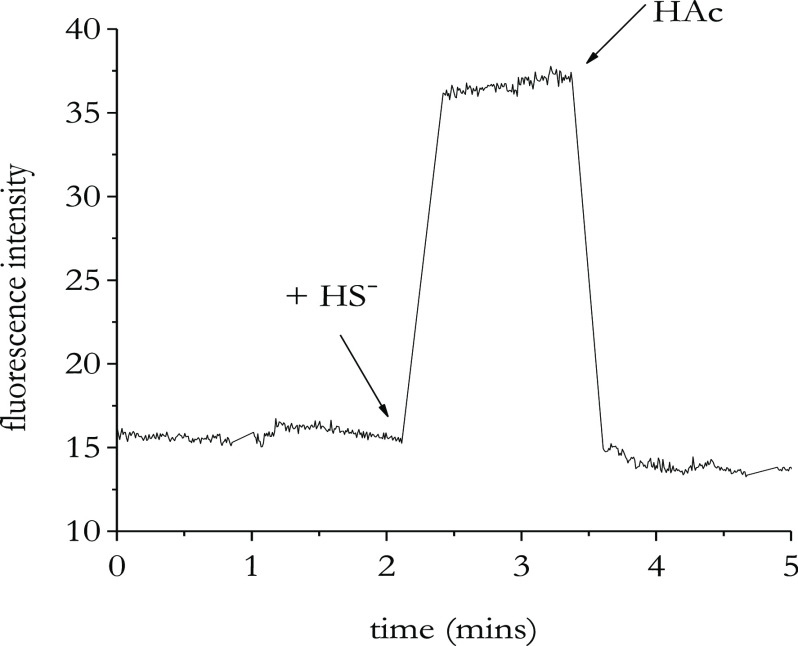
Emission spectrum of free complex **4** (λ_ex_ = 530 nm; λ_em_ = 630 nm), upon addition of 5 equiv of NaSH and upon addition of 10 equiv of acetic acid. [Complex **4**] = 1 × 10^–5^ M; [NaSH] = 5 × 10^–5^ M.

Still, in MQ water solution (see Figure 4), complex **4** undergoes a visible fluorescence enhancement in the presence of HS^–^. Next, we checked the reversibility of HS^–^ binding to the systems under investigation, in analogy with sensing constructs set up by us and others in the past.^14,27^ Indeed, in the case where HS^–^ coordination is acid-labile, the addition of a suitable proton source would result in a chemically reversible coordination of HS^–^. To test the reversibility of HS^–^ binding to complex **4**, we prepared the complex **4**-HS species *in situ* by adding 5 equiv of NaSH to the complex in water followed by an excess of acetic acid. As expected, the initial fluorescence intensity of complex **4**, which enhances upon the addition of HS^–^, was quenched when acetic acid was added. Figure 5 shows a typical time trace of a solution containing 10 μM of complex **4** when excited at 530 nm.

This fluorescence response suggests that the HS^–^ binding process is reversible, which is crucial for practical sensing applications.

The detection limit of complex **4** in MQ water solution was found in the micromolar range (Figure S18 in the SI).

To obtain an indication of the selectivity of these systems in the recognition of HS^–^ against potentially competing thiols (e.g., glutathione (GSH) and l-cysteine (l-Cys)), we monitored the fluorescence response of complex **4** in the presence of either GSH or l-Cys both in DMSO and in MQ water. In the presence of either GSH or l-Cys, we observed fluorescence responses different than that with HS^–^ (Figures S19 and S20), thus advising the selectivity of the sensing systems for HS^–^ detection.

The fluorescence switchings observed in the presence of NaSH for all the complexes under investigation, in addition to the red emissions, encouraged us to explore their possible applications as H_2_S sensing materials.

### Detection of HS^–^ and H_2_S by Complexes **1**–**4**

Following our screening of the optical features of the complexes under investigation, we explored their chromogenic chemosensing capability for the detection of HS^–^. In the presence of HS^–^, a color change, visible to the naked eye, occurred for all the complexes under investigation (see Figure S21) when dissolving the complexes both in DMSO and in acetone.

The above color change did not occur when adding GSH or l-cys to the DMSO or acetone solutions of complexes **1**–**4**, thus supporting the selectivity of the systems for HS^–^ detection already observed with the fluorescence experiments.

**Figure 6:**
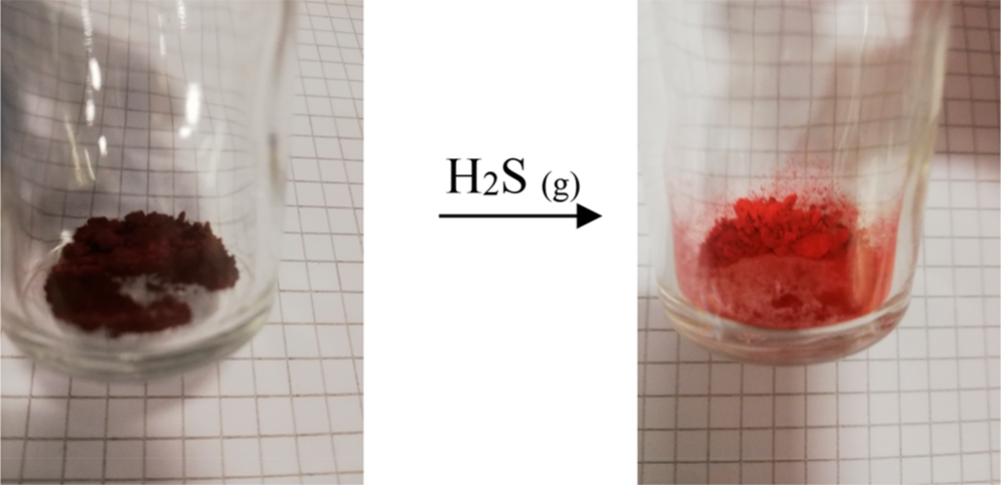
Real color images of complex **4** before (left image) and after (right image) flowing H_2_S gas.

As a further practical application, we also explored the use of complex **4** as a dosimeter for H_2_S gas. Dosimeters are irreversible devices, which progressively accumulate the dose, each time adding up the signal.^45,46^ For such a purpose, we flowed H_2_S gas on complex **4**, directly on the powder. As shown in Figure 6, an evident color change from dark purple to light orange occurred. Intriguingly, the longer we flowed the H_2_S gas on the powder, the brighter the orange color of the powder became.

Recently, dosimeters for H_2_S gas were set up by chemically treating filter paper test strips.^47^

**Figure 7:**
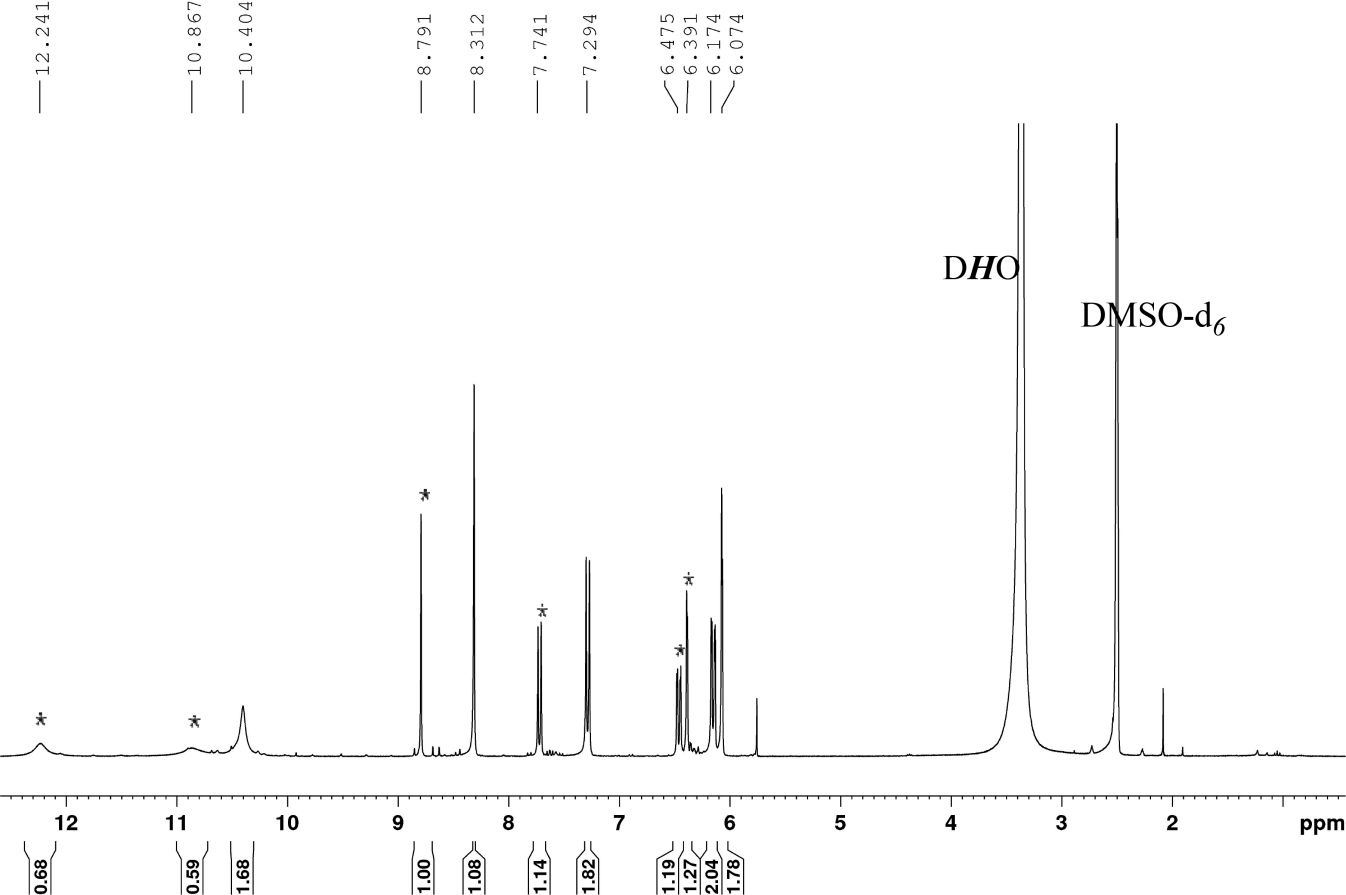
^1^H NMR spectrum of complex **4** in DMSO-*d*_6_ after bubbling H_2_S gas for 10 min. Peaks denoted with * belong to free ligand **4**, whereas the remaining peaks are those of free complex **4**.

To gain insights into the mechanism of the reaction occurring when complex **4** was exposed to an H_2_S atmosphere, the ^1^H NMR spectrum of the DMSO-*d*_6_ solution of the free complex after bubbling H_2_S gas directly in the NMR tube was obtained. Figure 7 displays the obtained spectrum which exhibits the signals of free complex **4** (see Figure S10) in addition to those of free ligand **4** (see Figure S11). This finding indicates that the interaction of complex **4** with H_2_S gas induces the displacement of the zinc center from the organic ligand, differently than that which we observed when complex **4** interacts with HS^–^ (see Figures S15 and S16). The color of the H_2_S-treated complex **4** closely resembles that of the free ligand **4**.

### Computational Study

**Figure 8:**
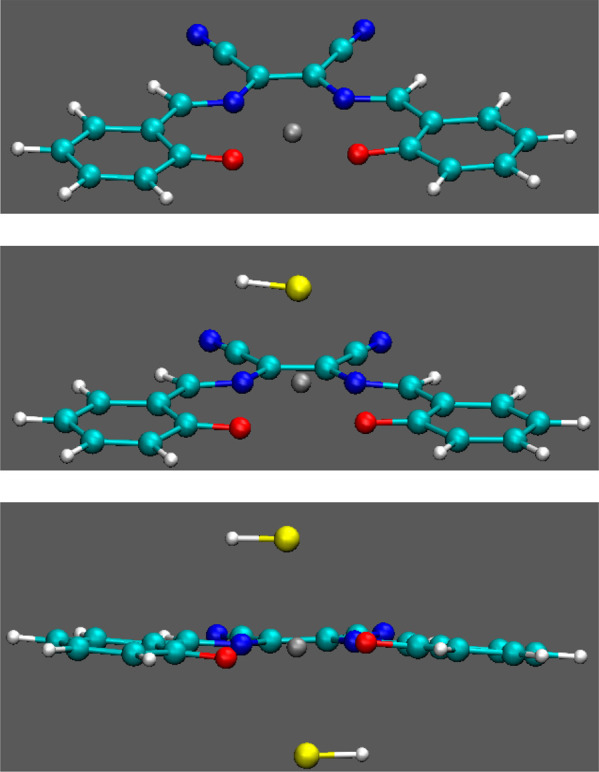
Optimized geometry for complex **1** (top) and its adduct with one (middle) or two (bottom) HS^–^.

In order to gain a deeper insight into the photophysical properties of compounds **1**–**4** and their adducts with HS^–^, we have performed a computational analysis on the time dependent density functional theory (TD-DFT) level, focusing on complexes **1** and **3**, which are representative of the whole class. Minimum energy geometries of **1** and **3** and of their HS^–^ adducts (also considering the possibility of multiple adducts) have been computed both for the ground state and for the first excited singlet states. The computed ground state optimum geometries of **1** and its HS^–^ adducts are shown in Figure 8.

Complex **1** exhibits the square planar nuclear configuration observed for Zn complexes (*C*_2*v*_ point group), with the metal atom in the plane of the ligand, see Figure 8. Upon coordination of a single HS^–^, the metal ion is slightly displaced out of the ligand plane and symmetry is lost, while upon coordination of two HS^–^, a *C*_2_ symmetry is predicted. The formation of the single adduct is predicted to be exoergonic (Δ*E* = −0.87 eV), whereas the formation of the double adduct is slightly endoergonic (Δ*E* = 0.15 eV), but the double adduct is a stable species, as confirmed by the eigenvalues of the computed Hessian matrix and by the fitting of experimental UV–vis absorption spectra, which predicts the formation of the double adduct at a high HS^–^ concentration, see SI Figures S23–S25 and Table S1.

A similar geometry has been also found for **3** and its HS^–^ complex, see Figure S22; however, complex **3** cannot coordinate more than a single HS^–^ ligand, the second one being moved away from the metal center during geometry optimization. Even for **3**, formation of the single adduct is predicted to be exoergonic, but the energy gain is smaller (Δ*E*= −0.77 eV). That result, together with the fact that the double adduct is not predicted to be a stable species can be traced back to the electron-donating effect of the N(CH_3_CH_2_)_2_ groups, which prevents Zn from accepting more electronic density by coordinating another HS^–^.

In the first excited singlet state (S_1_), the geometries of both **1** and **3** and of their HS^–^ adducts are only slightly distorted with respect to the ground state (S_0_), but *C*_2*v*_ symmetry of **1** and **3** is lowered to *C*_2_. Emission from S_1_ is predicted to be electric dipole allowed for both **1** and **3** and for their HS^–^ adducts. Computed vertical and adiabatic excitation energies are reported in Table 1, together with the oscillator strengths for the S_1_← S_0_ transitions. For **1**, three electric dipole allowed transitions are predicted in the spectral range 300–450 nm, in good agreement with the experimental absorption spectrum. The absorption frequencies are slightly overestimated (0.2–0.3 eV), but a meaningful comparison between predicted and observed absorption spectra would require band shape simulations, with the computations of Franck–Condon integrals,^48^ which is far beyond the qualitative purposes of the present computational analysis.

**Table 1:** Computed Vertical and Adiabatic Excitation Energies (eV) and Oscillator Strength for the S_1_ ← S_0_ Transitions

	vertical	adiabatic	oscillator strength
**1**	2.79	2.34	0.79
**1** + HS^–^	2.53	2.20	0.74
**1** + 2 HS^–^	2.32	2.03	0.64
**3**	2.63	2.34	1.47
**3** + HS^–^	2.48	2.19	1.26

For both **1** and **3**, and for their HS^–^ adducts, excitation to S_1_ correspond to the promotion of one electron from the HOMO to the LUMO (see Figures S26–S30). For all the investigated species, the LUMO is a nonbonding π MO with significant contributions from the π orbitals of the cyano groups, while the HOMO is a π level mainly localized on the central rings of the ligand, with, for **3**, significant contributions of the π orbitals of the nitrogen amine group, so that for **3**, the S_1_← S_0_ transition can be considered a charge transfer (CT) transition from the end-capping amines to the central cyano groups.

Since all the species’ emissions from S_1_ are electric dipole allowed transitions, the different behavior observed for **1** and **3** and for their HS^–^ complexes has to be related with the possible existence of nonradiative decay paths. We have thus investigated the energy location of the lowest triplet states, which could be responsible for the different fluorescence quantum yields of **1** and **3** and their HS^–^ adducts.

**Figure 9:**
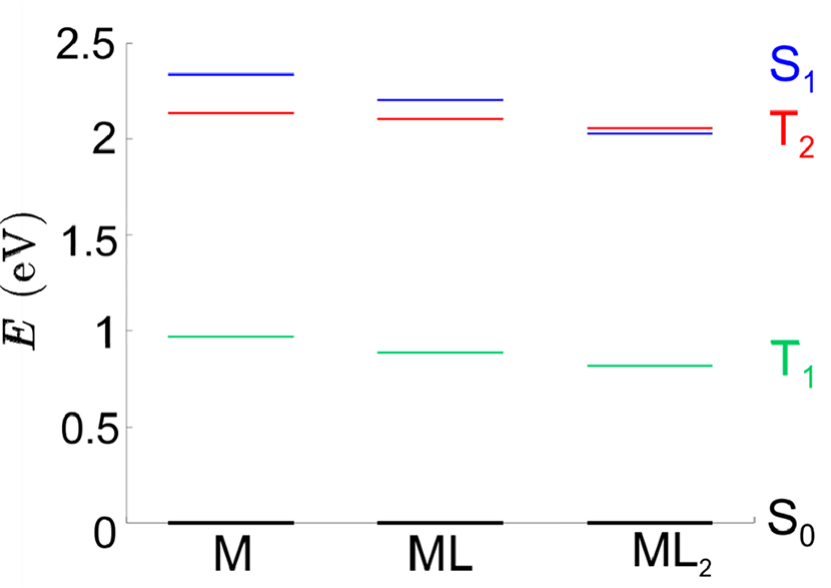
Computed energies (*E*, eV) of the ground state (S_0_), first excited singlet (S_1_), and two lowest energy triplet states (T_1_ and T_2_), for complex **1** (M), and its single (ML) and double (ML_2_) HS^–^ adducts.

The energy of the two lowest triplet states of complex **1** are reported in Figure 9; T_3_ lies always above in energy than S_1_ and therefore it should not be involved in nonradiative decay paths. The first triplet state T_1_ is significantly lower in energy than S_1_ for all the species, and therefore, based on the energy gap rule, the direct transition S_1_→ T_1_ should not be an efficient decay path. Vice versa, T_2_ is nearly degenerate with S_1_, being also strongerly coupled to it by spin–orbit couplings, reported in Table 2. Interestingly, the S_1_→ T_2_ transition is exoergonic for **1**, but it becomes slightly endoergonic (−0.03 eV) when two HS^–^ are coordinated (Figure 9). This suggests that quenching of fluorescence is quite possible in the isolated complex and its single HS^–^ adduct, whereas fluorescence emission is recovered when a large excess of HS^–^ is added in solution, in line with experimental observations (see Figure 3).

**Table 2:** Spin–Orbit Coupling Elements (cm^–1^) for **1** and **3** and Their Single Adducts

	spin–orbit coupling
**1**	38.65
**1** + HS^–^	68.88
**3**	13.97
**3** + HS^–^	197.11

As concerns complex **3**, the S_1_→ T_2_ transition is endoergonic, both for the isolated compound and for its single adduct with HS^–^. The first triplet state T_1_ is located too low in energy for being involved in efficient nonradiative decay pathways for both species, so that both **3** and its single HS^–^ adduct are predicted to emit from S_1_. While emission of **3** is indeed observed, fluorescence is quenched upon HS^–^ addition. This difference could be related to the high conformational flexibility of the single adducts of **1** and **3** (as suggested by inspection of Figures 8 and S22), which, as is well-known, can highly favor nonradiative decay to the ground state. Indeed, the single HS^–^ adducts of all the complexes studied here exhibit low fluorescence quantum yields. On the other hand, in the double adducts, if formed, conformational flexibility is lowered because of the high local dipole moments of the ligand, and thus fluorescence can be recovered. Since, as discussed above, **3** can only form the single adduct, a strong quenching of fluorescence is to be expected in the presence of HS^–^, as is indeed observed (Figure 3).

### Biological Assays in Living Cells

**Figure 10:**
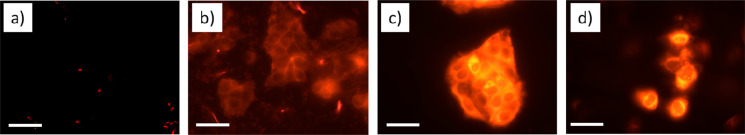
Fluorescence microscopy images of nontreated HepG2 cells (a) and HepG2 cells after 10 min of treatment with 120 μM complex **4** (b), 120 μM adduct (c), and 120 μM complex **4** + 250 μM NaSH (d). Magnification bar 50 μm.

The favorable optical features of complex **4** in the detection of HS^–^/H_2_S encouraged us to explore its potential in living cells. Before cell imaging experiments, we assessed the cytotoxicity of our probe via an MTT experiment. The MTT assay in HepG2 cells showed that complex **4** was not toxic under the experimental conditions tested (Figure S31). We next investigated the ability of complex **4** to visualize exogenous H_2_S in HepG2 cells. Therefore, we first incubated the cells for 10 min with our probe (120 μM) to explore whether complex **4** was able to permeate the cells. Figure 10 shows the cells after treatment with the sensing complex, and as evident, cells displayed a strong red fluorescence, thus indicating that the probe entered the cells (Figure 10b). We then incubated the cells with the complex **4**/HS adduct, which we had previously prepared in DMSO solution (in the same experimental conditions used for the fluorescence experiments in vitro). A clear fluorescence enhancement of the cells was observed (Figure 10c), indicating a good cell uptake of the adduct and its stability in cell culture conditions. To further investigate the ability of complex **4** to visualize exogenous H_2_S in living cells, we also compared the fluorescence of the cells incubated with complex **4** with that of the cells incubated with complex **4** and then treated with 250 μM NaSH (comparable with physiological concentrations) to allow the intracellular formation of HS^–^. Again, an evident fluorescence enhancement was observed (Figure 10d), demonstrating the capability of the probe to detect HS^–^ inside the cells directly. Additional images demonstrating the effects of the probe are reported in the SI (Figure S32).

To the best of our knowledge, application of zinc complexes as optical probes in biological studies is still limited;^44,49−52^ however, this is one of the first studies in which a zinc compound is used for imaging H_2_S in living cells.

## Conclusions

In conclusion, we prepared and studied the HS^–^/H_2_S reactivity of a suite of fluorescent zinc receptors in the framework of our ongoing studies aiming at further understanding the coordination chemistry of H_2_S/HS^–^ with bioinorganic targets. The present study was performed by using a variety of spectroscopic techniques (i.e., NMR, UV–vis and fluorescence) to gain independent evidence on the reactivity of HS^–^/H_2_S with the complexes under investigation. The fluorescence experiments provide a proof-of-principle that the title complexes may efficiently function as HS^–^ sensing constructs via a “coordinative-based” mechanism. A strong fluorescent enhancement is observed as a consequence of HS^–^ addition for complexes **1**, **2**, and **4**, whereas in the case of complex **3**, a quenching of the initial fluorescence intensity is seen. This difference and the overall fluorescence change have been assessed through a computational analysis, which related the fluorescence enhancement to a shift in the energy level of the excited triplet states in the double HS^–^ adduct, while the fluorescence quenching has been explained with the formation of single HS^–^ adducts, which highly favor nonradiative decay to the ground state.

Clearly visible color variations (visible to the naked-eye) occur when the complexes under investigation interact with HS^–^. A markedly different reactivity was found for complex **4** when it interacted with HS^–^ or with H_2_S. Preliminary biological experiments showed great potential in using this class of compounds as probes for the detection of H_2_S in living cells.

## Experimental Section

### Materials

All chemicals used for the synthetic work were obtained from Sigma-Aldrich or Strem Chemicals and were of reagent grade. They were used without further purification. Synthesis of complexes was performed by following literature procedures.^33,38,43^Also, ligand **4** was synthesized by literature procedures.^53^

### General

HR MALDI mass spectra were recorded using a Bruker solariX XR Fourier transform ion cyclotron resonance (FT-ICR) mass spectrometer (Bruker Daltonik GmbH, Bremen, Germany) equipped with a 7 T refrigerated actively shielded superconducting magnet (Bruker Biospin, Wissembourg, France). The samples were ionized in positive or negative ion mode using the MALDI ion source. The mass range was set to *m*/*z* 150–2000. The laser power was 15%, and 15 laser shots were used for each scan. Mass spectra were calibrated externally using a mix of peptide clusters in MALDI ionization positive ion mode. A linear calibration was applied. NMR spectra were recorded on a Bruker AVANCE 400 NMR instrument (^1^H NMR, 400.13 MHz; ^13^C NMR, 100.62 MHz) or on a 600 MHz spectrometer [600 (^1^H NMR) and 150 MHz (^13^C NMR)] using 5 mm o.d. NMR tubes. The chemical shifts were reported in δ (ppm) referenced to SiMe4. Typically, 5 mg of the complex in 0.5 mL of the solvent was used for each experiment.

#### Characterization of Ligand **4**

^1^H NMR [400 MHz, DMSO-*d*_6_]: δ 12.23 (s, 2H, O***H***), (10.87 (s, 2H, O***H***), 8.80 (s, 2H, C***H***=N), 7.74 (d, *J* = 8.7 Hz, 2H, ***H*** aromatic), 6.50 (d, *J* = 8.7 Hz, 2H, ***H*** aromatic), 6.40 (s, 2H, ***H*** aromatic).

#### Characterization of Complex **1**

MS (MALDI FT-ICR THF), *m*/*z* (%) calculated: 379.016. Experimental: 379.017 [complex **1**+H]^+^. ^1^H NMR [400 MHz, DMSO-*d*_6_]: δ 8.56 (s, 2H, C***H***=N), 7.48 (dd, *J* = 8.1 Hz, ^4^*J* = 1.8 Hz, 2H, ***H*** aromatic), 7.37 (dt, *J* = 7.6 Hz, ^4^*J* = 1.8 Hz, 2H, ***H*** aromatic), 6.77 (d, *J* = 8.6 Hz, 2H, ***H*** aromatic), 6.57 (t, *J* = 7.4 Hz, 2H, ***H*** aromatic). Emission (DMSO, λ_exc_ = 560 nm), λ_max_, nm (quantum yield, Φ_F_): 630 nm (0.01).

#### Characterization of Complex **2**

MS (MALDI FT-ICR THF), *m*/*z* (%) calculated: 439.037. Experimental: 439.042 [complex **2**+H]^+^. ^1^H NMR [400 MHz, DMSO-*d*_6_]: δ 8.54 (s, 2H, C***H***=N), 7.02 (d, *J* = 8.2 Hz, 2H, ***H*** aromatic), 6.94 (d, *J* = 7.4 Hz, 2H, ***H*** aromatic), 6.48 (t, *J* = 7.8 Hz, 2H, ***H*** aromatic), 3.78 (s, 6H, OC***H***_3_). Emission (DMSO, λ_exc_ = 410 nm), λ_max_, nm (quantum yield, Φ_F_): 500 nm (0.07).

#### Characterization of Complex **3**

MS (MALDI FT-ICR THF), *m*/*z* (%) calculated: 520.155. Experimental: 520.159 [complex **3**]^+^. ^1^H NMR [400 MHz, DMSO-*d*_6_]: δ 8.13 (s, 2H, C***H***=N), 7.15 (d, *J* = 9.2 Hz, 2H, ***H*** aromatic), 6.21 (d, *J* = 9.2 Hz, 2H, ***H*** aromatic), 5.83 (s, 2H, ***H*** aromatic), 3.40 (q, *J* = 6.7 Hz, 8H, NC***H***_2_CH_3_), 1.14 (t, *J* = 6.7 Hz, 12H, NCH_2_C***H***_3_). Emission (DMSO, λ_exc_ = 440 nm), λ_max_, nm (quantum yield, Φ_F_): 645 nm (0.4).

#### Characterization of Complex **4**

MS (MALDI FT-ICR THF), *m*/*z* (%) calculated: 411.006. Experimental: 411.006 (100) [complex **4**+H]^+^. ^1^H NMR [400 MHz, DMSO-*d*_6_]: δ 10.40 (s, 2H, O***H***), 8.33 (s, 2H, C***H***=N), 7.32 (d, *J* = 8.8 Hz, 2H, ***H*** aromatic), 6.18 (dd, *J* = 8.8 Hz, ^4^*J* = 2.2 Hz, 2H, ***H*** aromatic), 6.08 (d, ^4^*J* = 2.2 Hz, 2H, ***H*** aromatic). Emission (DMSO, λ_exc_ = 570 nm), λ_max_, nm (quantum yield, Φ_F_): 620 nm (0.2).

#### Absorbance and Fluorescence Measurements

Absorption spectra were recorded on a Cary-50 Spectrophotometer, using a 1 cm quartz cuvette (Hellma Benelux bv, Rijswijk, Netherlands) and a slit-width equivalent to a bandwidth of 5 nm. Fluorescence spectra were measured on a Cary Eclipse Spectrophotometer in a 10 × 10 mm^2^ airtight quartz fluorescence cuvette (Hellma Benelux bv, Rijswijk, Netherlands) with an emission band-pass of 10 nm and an excitation band-pass of 5 nm. Both absorption and fluorescence measurements were performed either in DMSO or in Milliq water solutions at 25 °C. Fluorescence emission spectra were registered by exciting the samples at a specific wavelength (as stated in the figure captions).

Fluorescence quantum yield (Φ_F_) values were measured in optically diluted solutions using as standards the commercial dyes Cy5 NHS (Φ_F_ = 0.28 in Milli-Q water) in the case of complexes **1**, **3**, and **4** and Cy3 NHS (Φ_F_ = 0.15 in Milli-Q water) in the case of complex **2**, according to the equation^54^
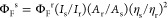
where indexes s and r denote the sample and reference, respectively. *I* stands for the integrated emission intensity. *A* is the absorbance at the excitation wavelength, and η is the refractive index of the solvent. The optical density of complexes **1**–**4** and standards was kept below 0.1. The uncertainty in the determination of Φ_F_ is ±15%.

### NMR Characterization of the Complexes **1**–**4** upon Addition of HS^–^

The NMR tube was charged with the free complex solutions in DMSO-*d*_6_; then NaSH solid or in solution (to the end concentrations specified in the figure captions) was added and the spectra registered.

### H_2_S Dosimeter Experiments

In the case of complex **4**, the H_2_S dosimeter experiments were performed as follows: the vial was filled with the powder sample and closed, then an H_2_S gas flow was maintained on top of the powder. The experiment ended when no changes in the color of the powder could be detected.

### Cell Culture

HepG2 cells (Human hepatocellular liver carcinoma cell line) were grown in Minimum Essential Medium (MEM) supplemented with 10% fetal bovine serum (FBS), 2 mM glutamine, 1 mM nonessential amino acids, and 1% antibiotics (penicillin/streptomycin, 100 U/mL). Cells were maintained in a humidified incubator at 37 °C, in 5% CO_2_/95% air. Then, 2 × 10^5^ cells were seeded on 12 mm glass coverslips in a culture dish 1 day before imaging.

### MTT Assay

Cell viability was analyzed by 3-(4,5-dimethylthiazol-2-yl)-2,5-diphenyltetrazolium bromide (MTT; Sigma-Aldrich) assay. Then, 1.5 × 10^4^ cells were seeded in each well of a 96-multiwell plate. Twenty-four hours after cell seeding, HepG2 cells were incubated with increasing concentrations of complex **4** (0.05, 0.5, 5, and 50 μg/mL) obtained by diluting complex **4** stock solution (1 mg/mL in DMSO) in cell culture medium. After 24 h of incubation with complex **4** solutions, the MTT reagent was added to the cell media of each sample (final concentration 0.125 mg/mL) and incubated for 1 h at 37 °C. The resulting formazan crystals were dissolved in DMSO. Absorbance was measured at 570 and 690 nm wavelengths by a multiplate reader, and raw data were normalized to nontreated cells (considered 100%) to calculate cell viability percentage. Data were reported as mean ± standard deviation (*n* = 8).

### Fluorescence Imaging

To verify the loading of the probe and the adduct, HepG2 cells were incubated with 120 μM complex **4** and 120 μM adduct diluted in HBSS for 10 min at 37 °C. The adduct was preformed by adding complex **4** to NaSH. After incubation, cells were rinsed to remove the excess complex **4** and adduct. Probe- and adduct-loaded cells were observed by an epifluorescence microscope (Zeiss) at a 543 nm excitation wavelength and a 40× oil-immersion objective. Only probe-loaded cells were further treated with exogenous NaSH (250 μM in HBSS) for 10 min and then observed with the microscope to test the capability of complex **4** to monitor the intracellular increase of H_2_S.

### Computational Details

All electronic computations have been carried out at the density functional level of theory using the range separated hybrid functional CAM-B3LYP with TZVP basis set as implemented in the Gaussian package (G09).^55^ That combination of functional and basis set has been chosen because it leads to accurate predictions, as discussed in previous works.^56,57^ Time dependent DFT (TD-DFT) has been employed for treating all excited states. Spin–orbit coupling elements have been computed by PySOC code.^58^ Effects due to solvent polarization were included by the polarizable continuum model (PCM).^59^

## Supporting Information Available

MALDI spectra of complexes **1**–**4**; ^1^H NMR spectra of complexes **1**–**4** before and after addition of NaSH; fluorescence spectra of complex **4** in DMSO and in MQ water in the presence of NaOH, GSH, and l-Cys; optimized geometries for complex **3**; experimental and simulated absorption spectra of complex **1**; predicted and experimental molar absorbances of complex **1** and its adducts; HOMO and LUMO of complexes **1** and **3** and of their HS^–^ adducts; cytotoxicity evaluation of complex **4** in the HepG2 cell line by MTT assay; additional fluorescence microscopy images of HepG2 cells; Cartesian coordinates of all the optimized structures; photophysical properties of complexes 1-4 and of their HS^–^ adducts (PDF)
