# Supplementary material for: Fluorescent salen-type Zn(II) Complexes As Probes for Detecting Hydrogen Sulfide and Its Anion: Bioimaging Applications
Source: Inorg Chem. 2020 Oct 13;59(21):15977–86. doi: 10.1021/acs.inorgchem.0c02499 (PMC8015222; doi:10.1021/acs.inorgchem.0c02499)
Supplement: Supplementary file 1 — ic0c02499_si_001.pdf [file ic0c02499_si_001.pdf]

# Fluorescent *salen*-type Zn(II) complexes as probes for detecting hydrogen sulfide and its anion: bio-imaging applications.

Maria Strianese*, Daniela Guarnieri, Marina Lamberti, Alessandro Landi, Andrea Peluso and Claudio Pellecchia.

Dipartimento di Chimica e Biologia "Adolfo Zambelli", Università degli Studi di Salerno, Via Giovanni Paolo II, 132, 84084 Fisciano (SA) Italy
*Corresponding author. E-mail: mstriane@unisa.it

## Supporting information.

**Contents:**

Synthetic procedures for complexes **1-4** 3

Figure S1, MALDI spectrum of complex **1** 4

Figure S2, MALDI spectrum of complex **2** 5

Figure S3, Enlargement of the MALDI spectrum of complex **2** 6

Figure S4, MALDI spectrum of complex **3** 7

Figure S5, Enlargement of the MALDI spectrum of complex **3** 8

Figure S6, MALDI spectrum of complex **4** 9

Figure S7, $^1$H NMR spectrum of complex **1** in DMSO-$d_6$ 10

Figure S8, $^1$H NMR spectrum of complex **2** in DMSO-$d_6$ 11

Figure S9, $^1$H NMR spectrum of complex **3** in DMSO-$d_6$ 12

Figure S10, $^1$H NMR spectrum of complex **4** in DMSO-$d_6$ 13

Figure S11, $^1$H NMR spectrum of ligand **4** in DMSO-$d_6$ 14

Figure S12, $^1$H NMR spectrum of complex **1** in DMSO-$d_6$ upon addition of NaHS 15

Figure S13, $^1$H NMR spectrum of complex **2** in DMSO-$d_6$ upon addition of NaHS 16

Figure S14, $^1$H NMR spectrum of complex **3** in DMSO-$d_6$ upon addition of NaHS 17

Figure S15, $^1$H NMR spectrum of complex **4** in DMSO-$d_6$ upon addition of NaHS 18

Figure S16, NOESY spectrum of complex **4** in DMSO-$d_6$ upon addition of NaHS 19

Figure S17, Fluorescence spectra of complex **4** in DMSO before and after addtion of NaOH 20

Figure S18, Detection limit experiments 21

Figure S19, Fluorescence spectra of complex **4** in DMSO before and after addition of GSH and L-Cys 22

Figure S20, Fluorescence spectra of complex **4** in MQ water 23

Figure S21, Images of solutions of complexes **1-4** after $HS^-$ treatment 24

Figure S22, Optimized geometries for complex **3** 25

Figure S23, Electronic absorption spectra of complex **1** 27

Figure S24, Experimental and simulated spectra of complex **1** 28

Figure S25, Predicted and experimental molar absorbances of complex **1** and its adducts 29

Table S1, Relative abundances for complex **1** of the species M, ML and $ML_2$ for different $HS^-$ concentrations 30

Figure S26, HOMO and LUMO of complex **1** 31

Figure S27, HOMO and LUMO of complex **1** + $HS^-$ 31

Figure S28, HOMO and LUMO of complex **1** + 2 $HS^-$ 31

Figure S29, HOMO and LUMO of complex **3** 32

Figure S30, HOMO and LUMO of complex **3** + $HS^-$ 32

Figure S31, Cytotoxicity evaluation of complex **4** in HepG2 cell line by MTT assay 33

Figure S32, Additional fluorescence microscopy images of HepG2 cells after 10 min treatment with complex **4**, adduct and complex **4** + 250 µM NaSH 34

Cartesian coordinates of all the optimized structures 35

Table S2, Photophysical properties 36

Complex **1**: A mixture of salicylaldehyde (0.54 mL, 5.8 mmol), diaminomaleonitrile (0.31 g, 2.9 mmol) and $Zn(CH_3COO)_2*2H_2O$ (0.64 g, 2.9 mmol) in 100 mL of absolute ethanol was left under stirring for 2 days at 70 °C. A purple solid was recovered by filtration, this was then washed with cold methanol and dried under vacuum (yield 75 %).

Complex **2**: A mixture of *o*-vanillin (0.88 g, 5.8 mmol), diaminomaleonitrile (0.31 g, 2.9 mmol) and $Zn(CH_3COO)_2*2H_2O$ (0.64 g, 2.9 mmol) in 100mL of DMF was left under stirring overnight at 25 °C. A purple precipitate was recovered by filtration, this was then washed with cold methanol and dried under vacuum (yield 70 %).

Complex **3**: A mixture of 4-(diethylamino)salicylaldehyde (386.5 mg, 2 mmol) and diaminomaleonitrile (108 mg, 1 mmol) in 50 mL of hot absolute ethanol was added to $Zn(CH_3COO)_2*2H_2O$ (219.5 mg, 1 mmol) in 150 mL of hot absolute ethanol and stirred for 2 days at 70 °C. The resulting red mixture was then cooled in a freezer. A dark blue precipitate was recovered by filtration, this was then washed with cold methanol and dried under vacuum (yield 50%).

Complex **4**: A mixture of 2,4-dihydroxybenzaldehyde (0.801 g, 5.8 mmol), diaminomaleonitrile (0.31 g, 2.9 mmol), and $Zn(CH_3COO)_2*2H_2O$ (0.64 g, 2.9 mmol) in 100 mL of DMF was left under stirring over night at 25 °C. A purple precipitate was recovered by filtration, this was then washed with cold methanol and dried under vacuum (yield 75 %).

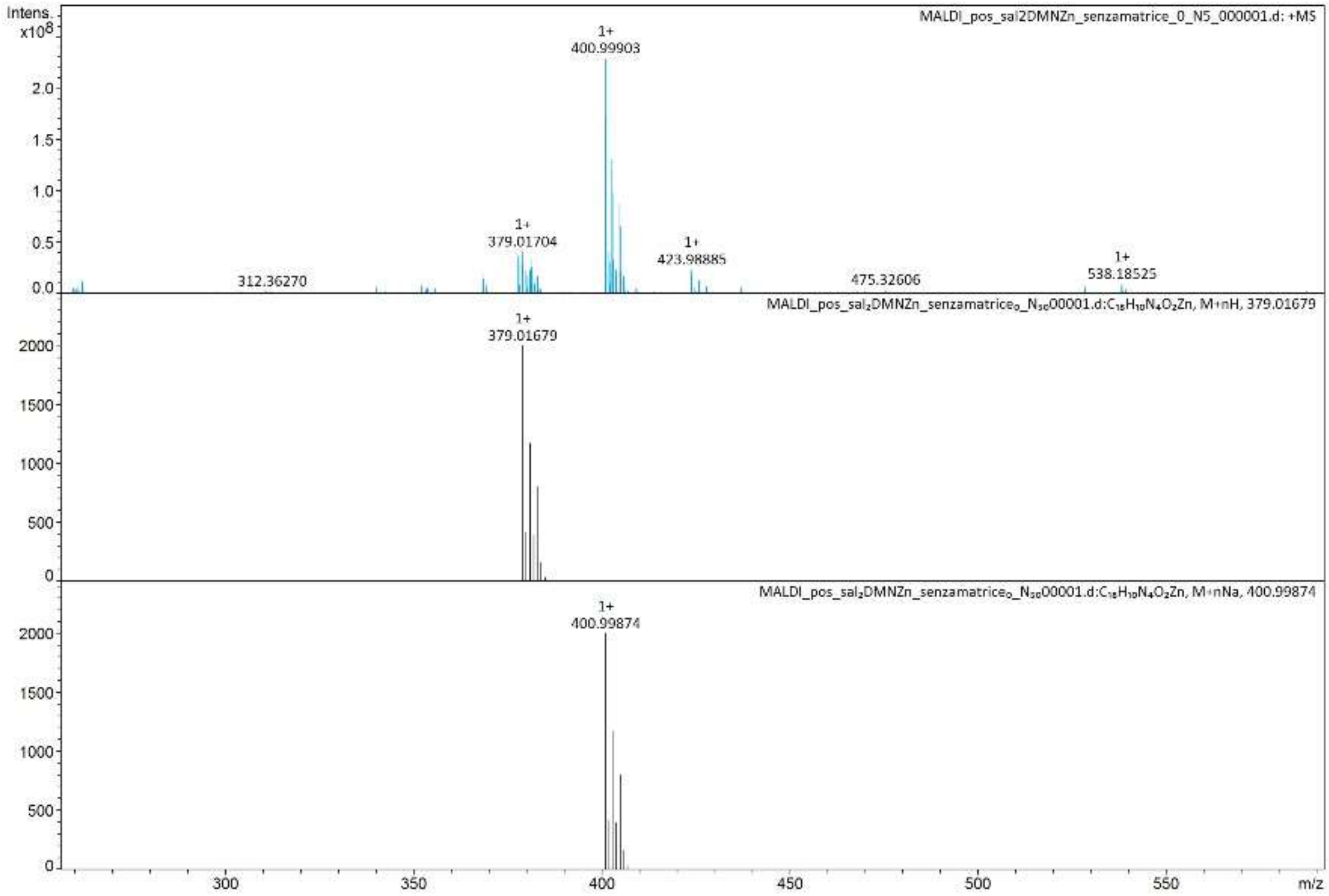


**Figure S1.** MALDI spectrum of complex **1** in THF. The upper trace is the experimental trace whereas the lowers are the theoretical ones.

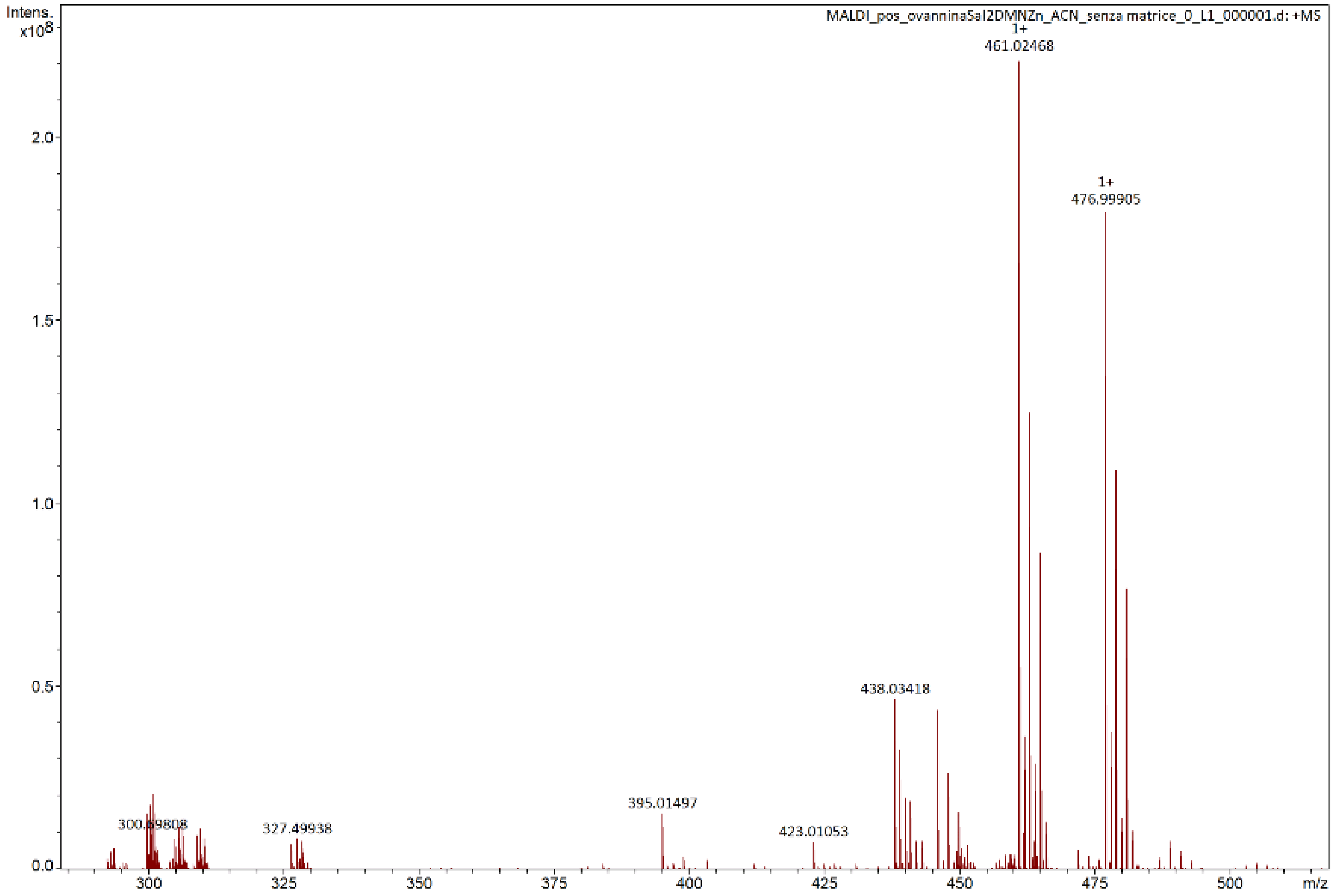


**Figure S2.** Full MALDI spectrum of complex **2** in THF.

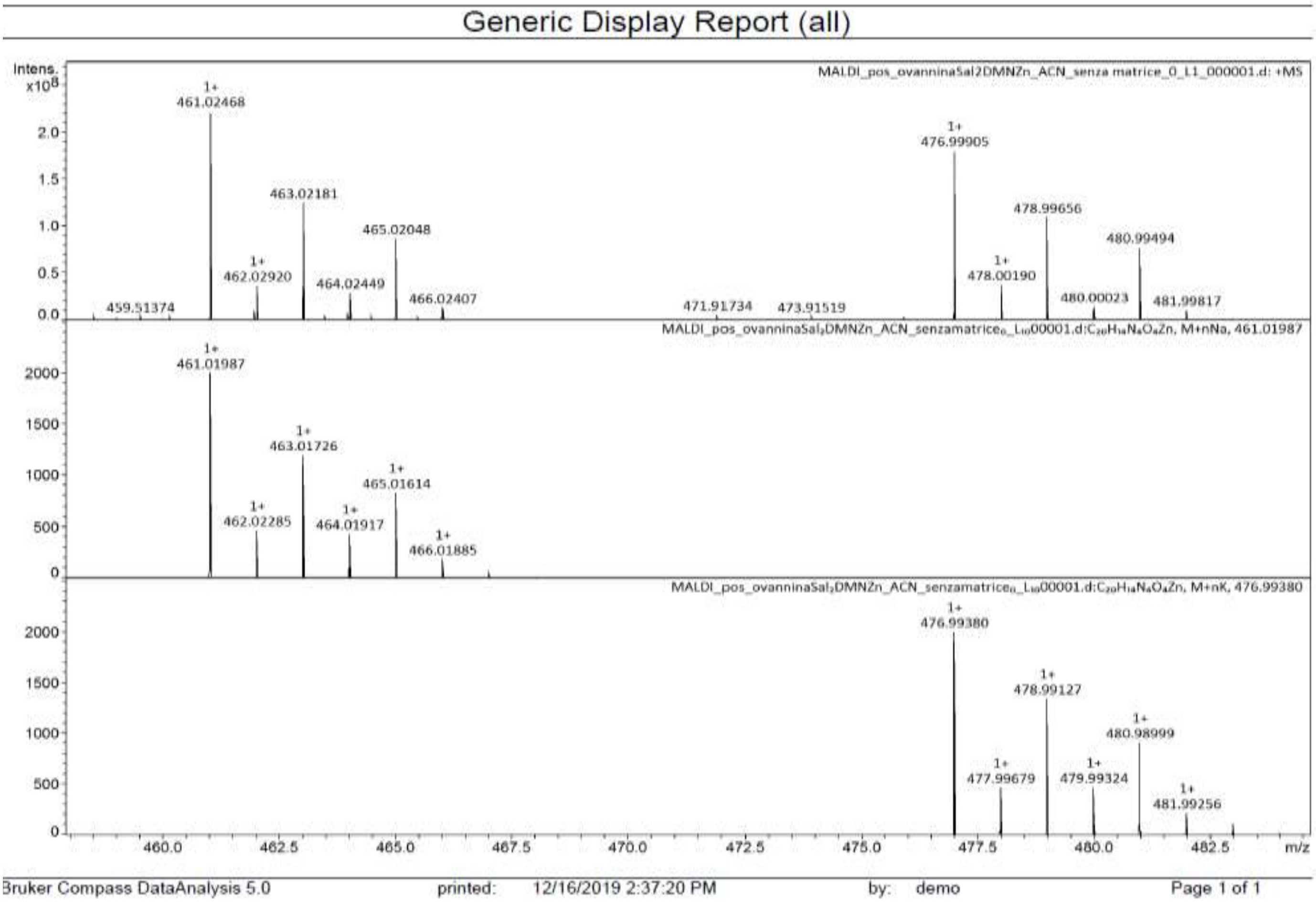


**Figure S3.** Enlargement of a region of the MALDI spectrum of complex **2** in THF. The upper trace is the experimental trace whereas the lowers are the theoretical ones.

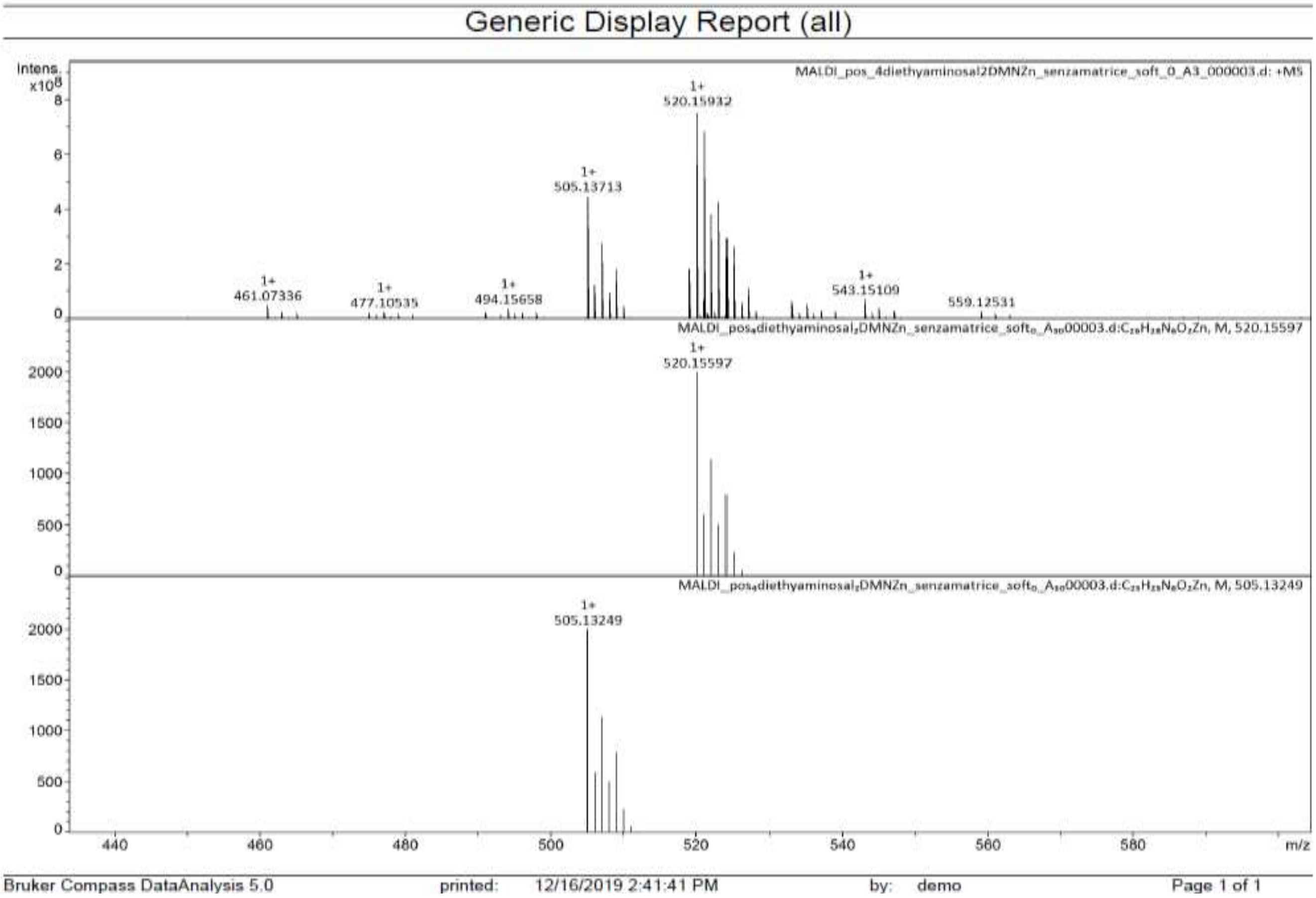


**Figure S4.** MALDI spectrum of complex **3** in THF.

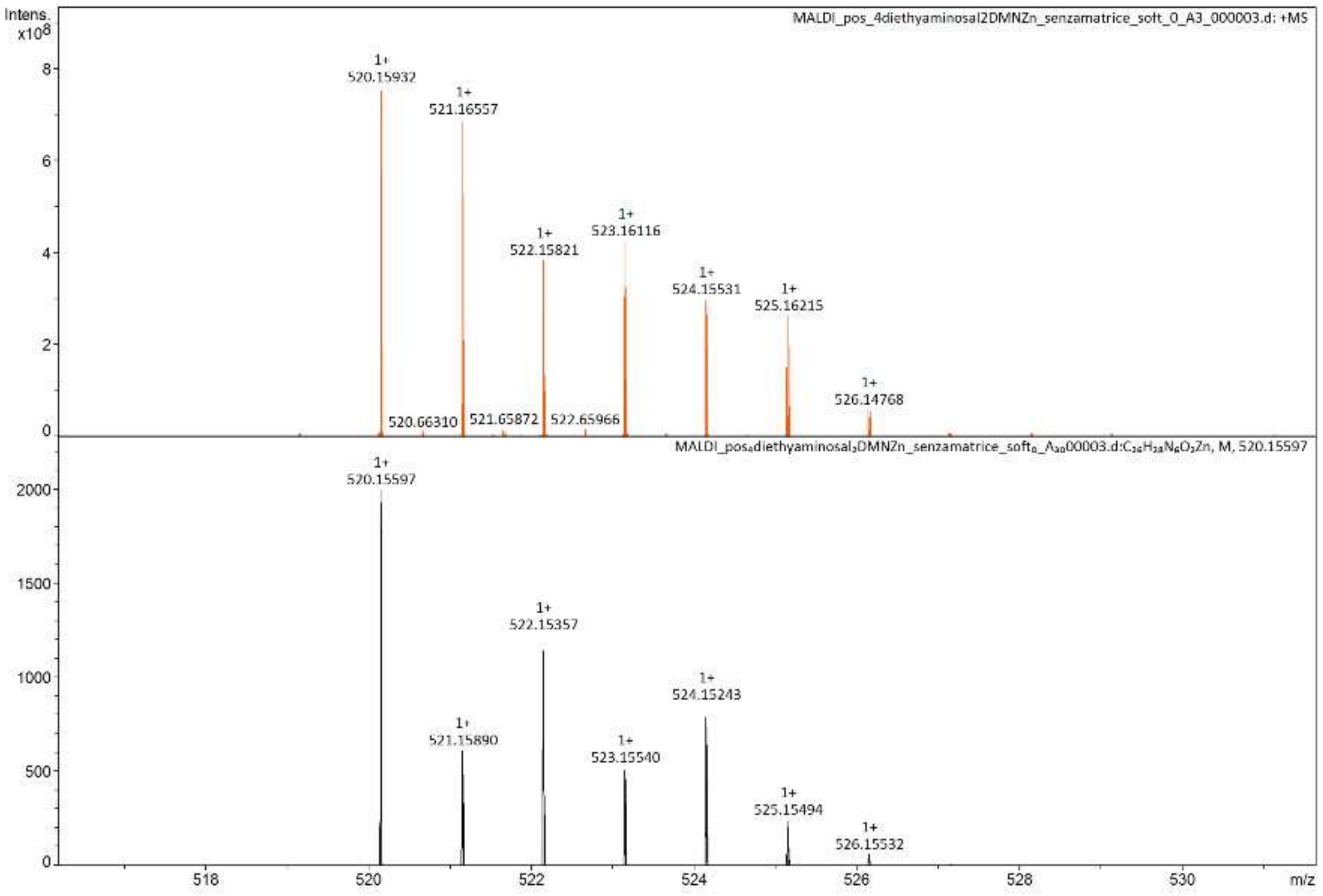


**Figure S5.** Enlargement of a region of the MALDI spectrum of complex **3** in THF. The upper trace is the experimental trace whereas the lower is the theoretical one.

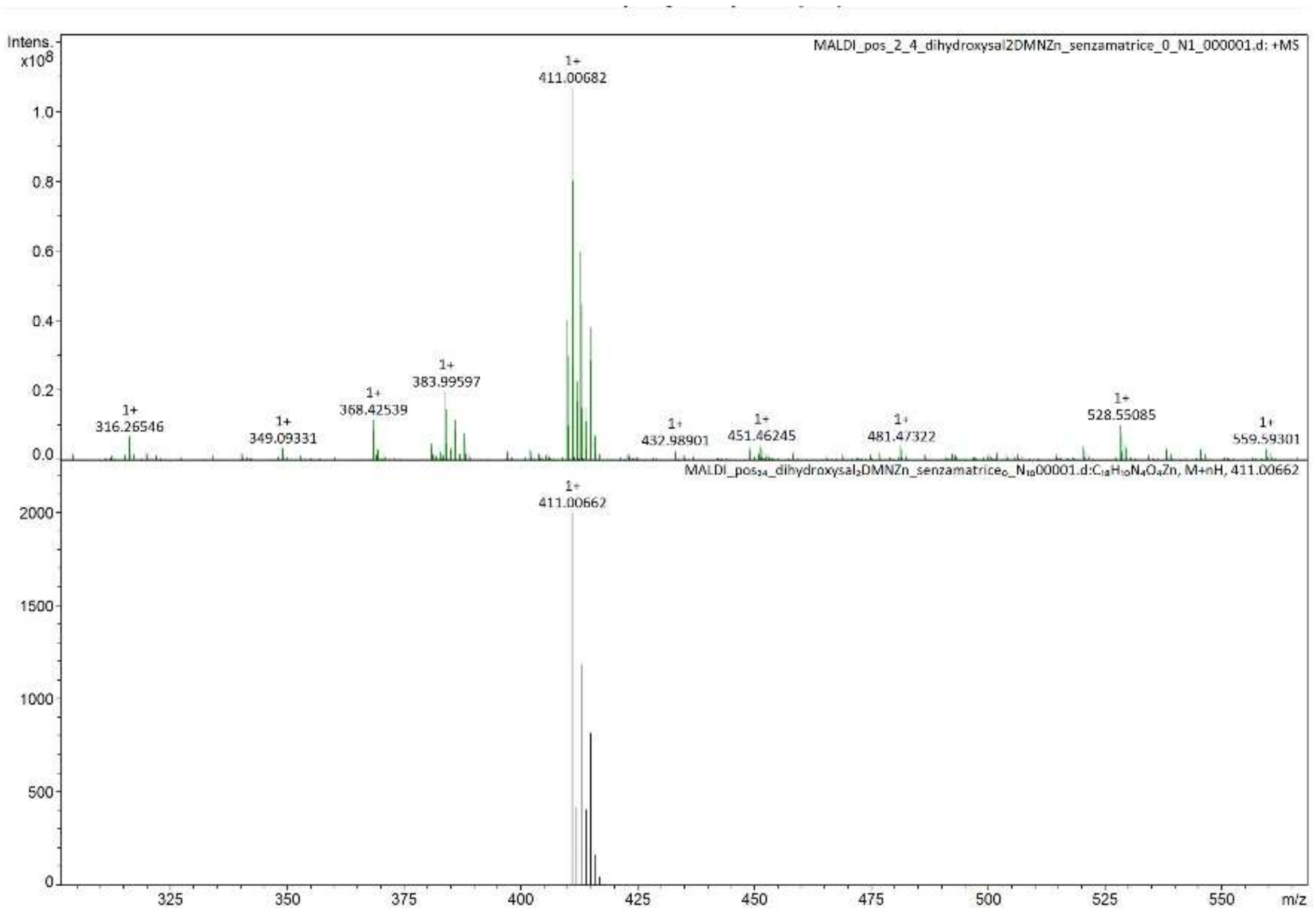


**Figure S6.** MALDI spectrum of complex **4** in THF.

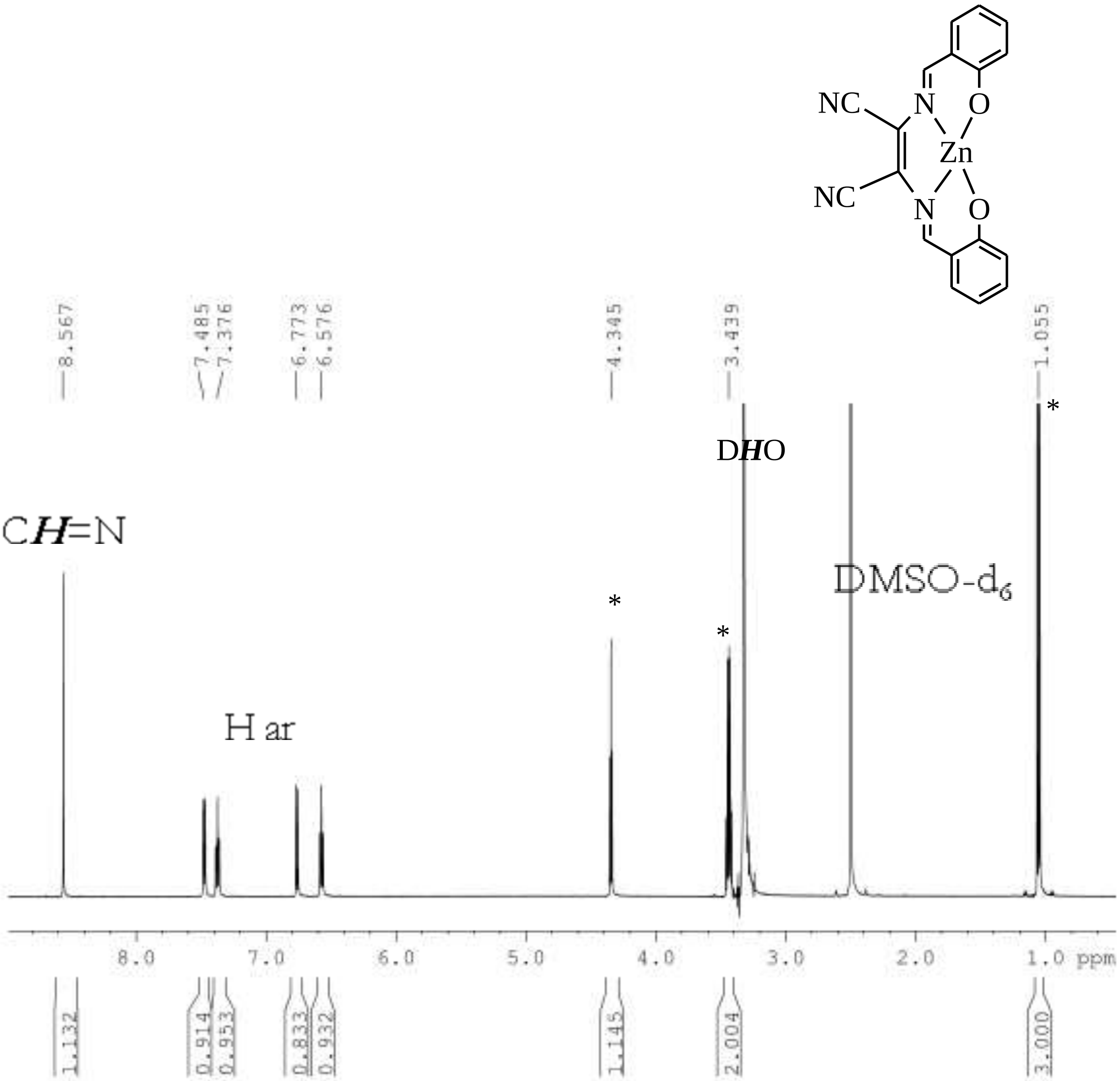


**Figure S7**. $^{1}H$ NMR spectrum of complex **1** in DMSO-$d_6$. [complex **1**] = $50\times10^{-3}$ M. Peaks denoted with * correspond to ethanol used for the synthesis.

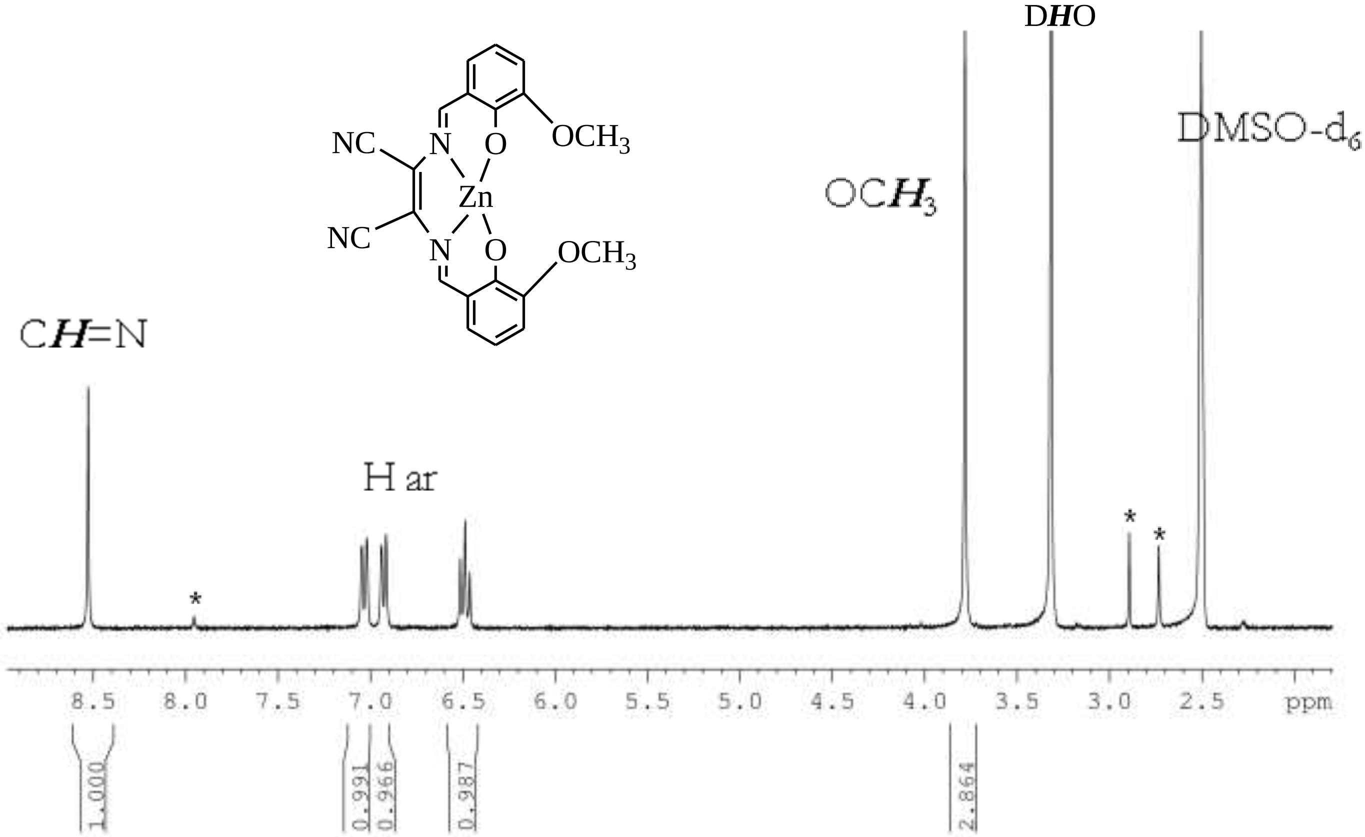


**Figure S8**. $^{1}$H NMR spectrum of complex **2** in DMSO-$d_6$ . [complex **2**] = $50\times10^{-3}$ M. Peaks denoted with * correspond to DMF used for synthesis.

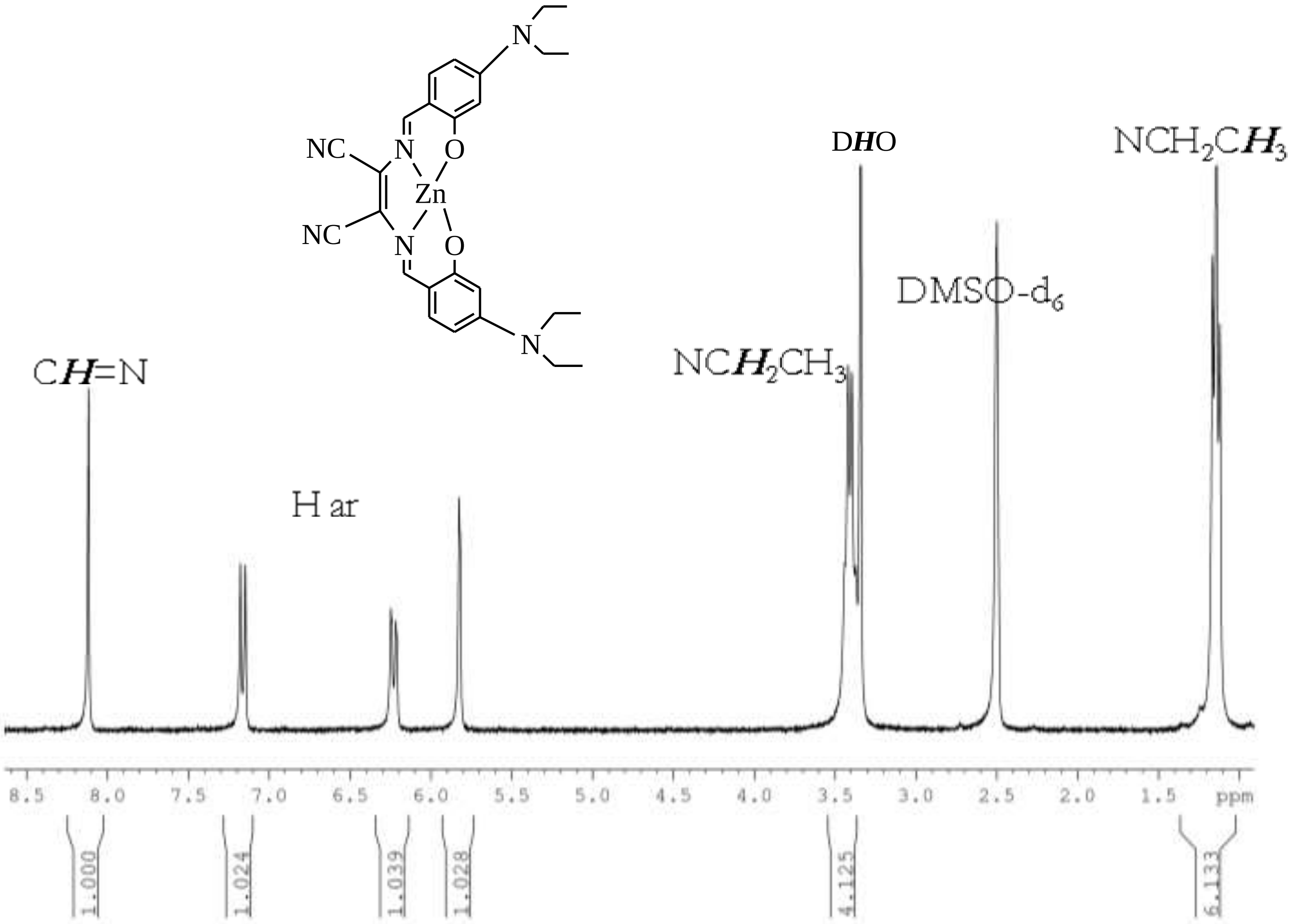


**Figure S9**. $^{1}$H NMR spectrum of complex **3** in DMSO-$d_6$ . [complex **3**] = $50\times10^{-3}$ M.

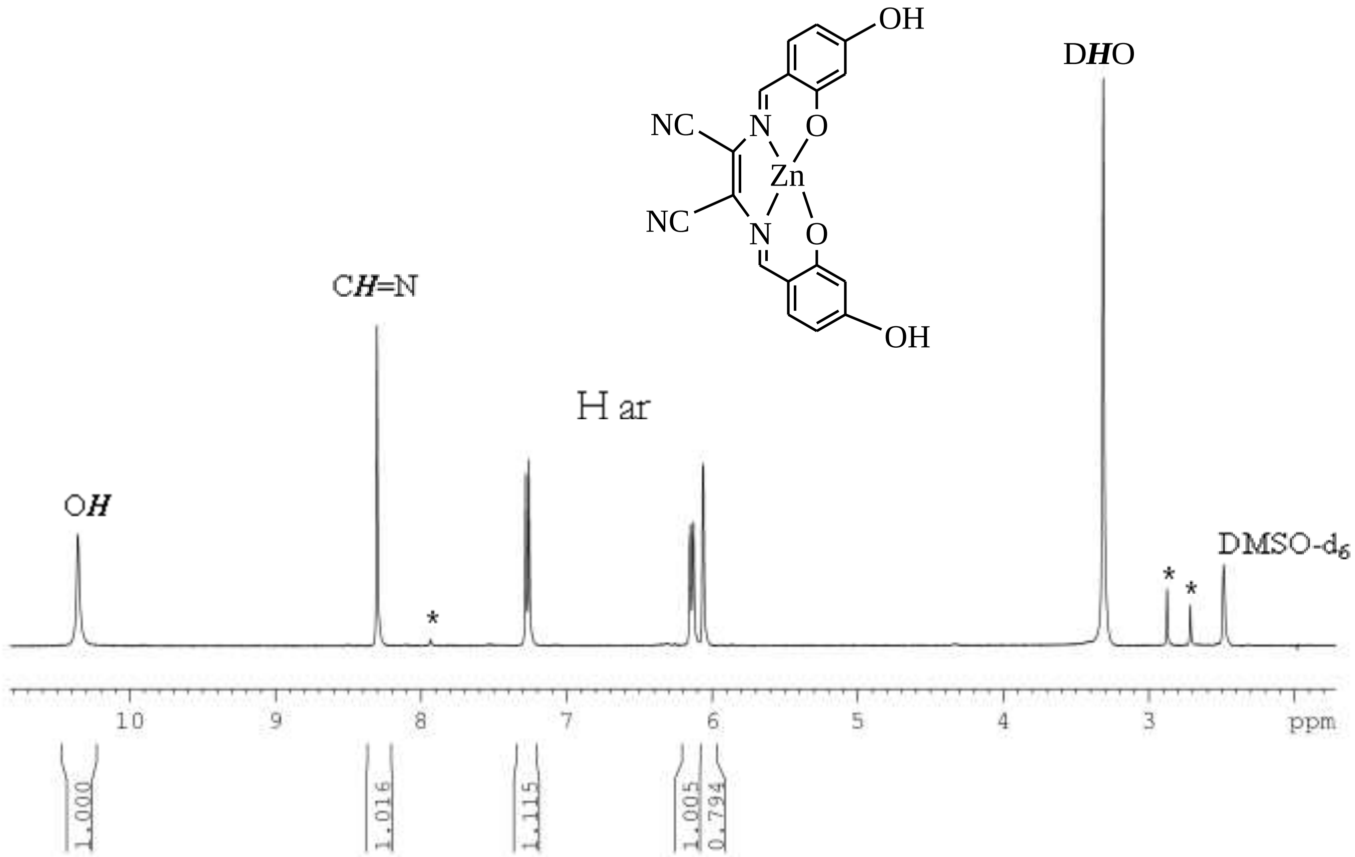


**Figure S10**. $^{1}$H NMR spectrum of complex **4** in DMSO-d$_6$. [complex **4**] = 50×10$^{-3}$ M. Peaks denoted with * correspond to DMF used for synthesis.

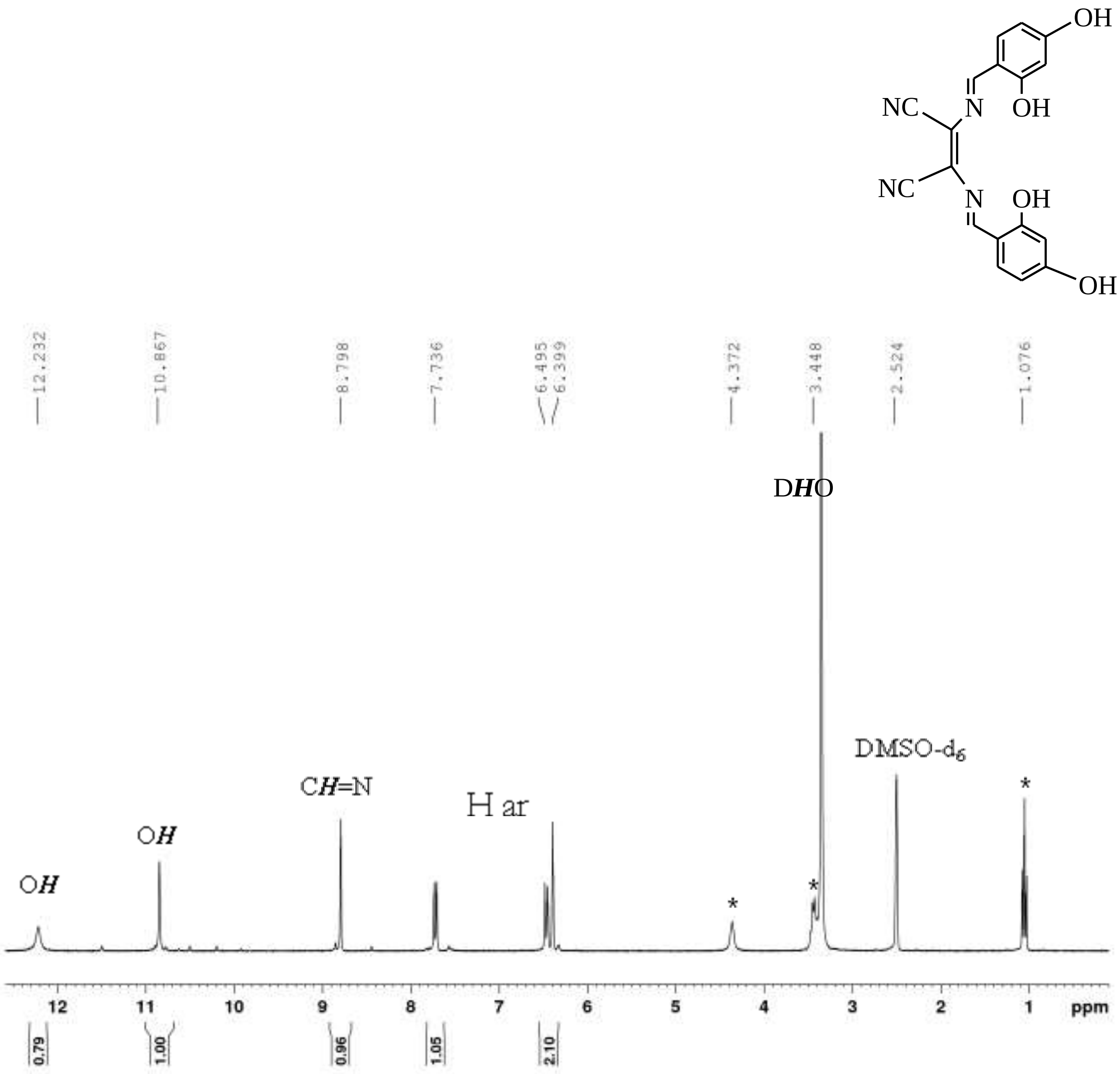


**Figure S11**. $^{1}$H NMR spectrum of ligand **4** in DMSO-$d_6$. [ligand **4**] = $50\times10^{-3}$ M. Peaks denoted with * correspond to ethanol used for synthesis.

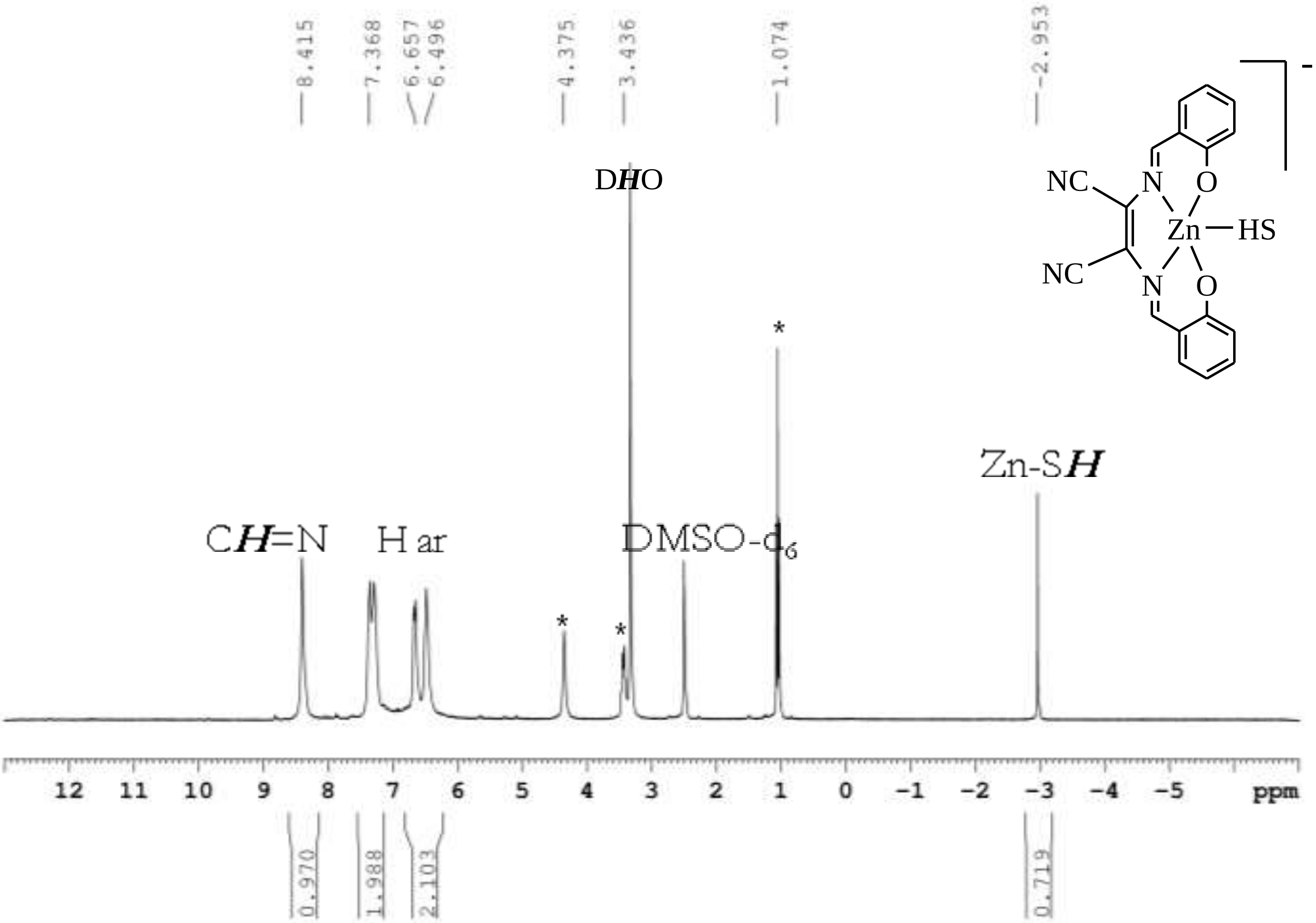


**Figure S12.** $^{1}$H NMR spectrum of complex **1** in DMSO-$d_6$ after the addition of an excess of $HS^{-}$. Peaks denoted with *correspond to ethanol used for the synthesis. [complex **1**] = $5\times10^{-2}$ M; [NaSH] = 0.25 M.

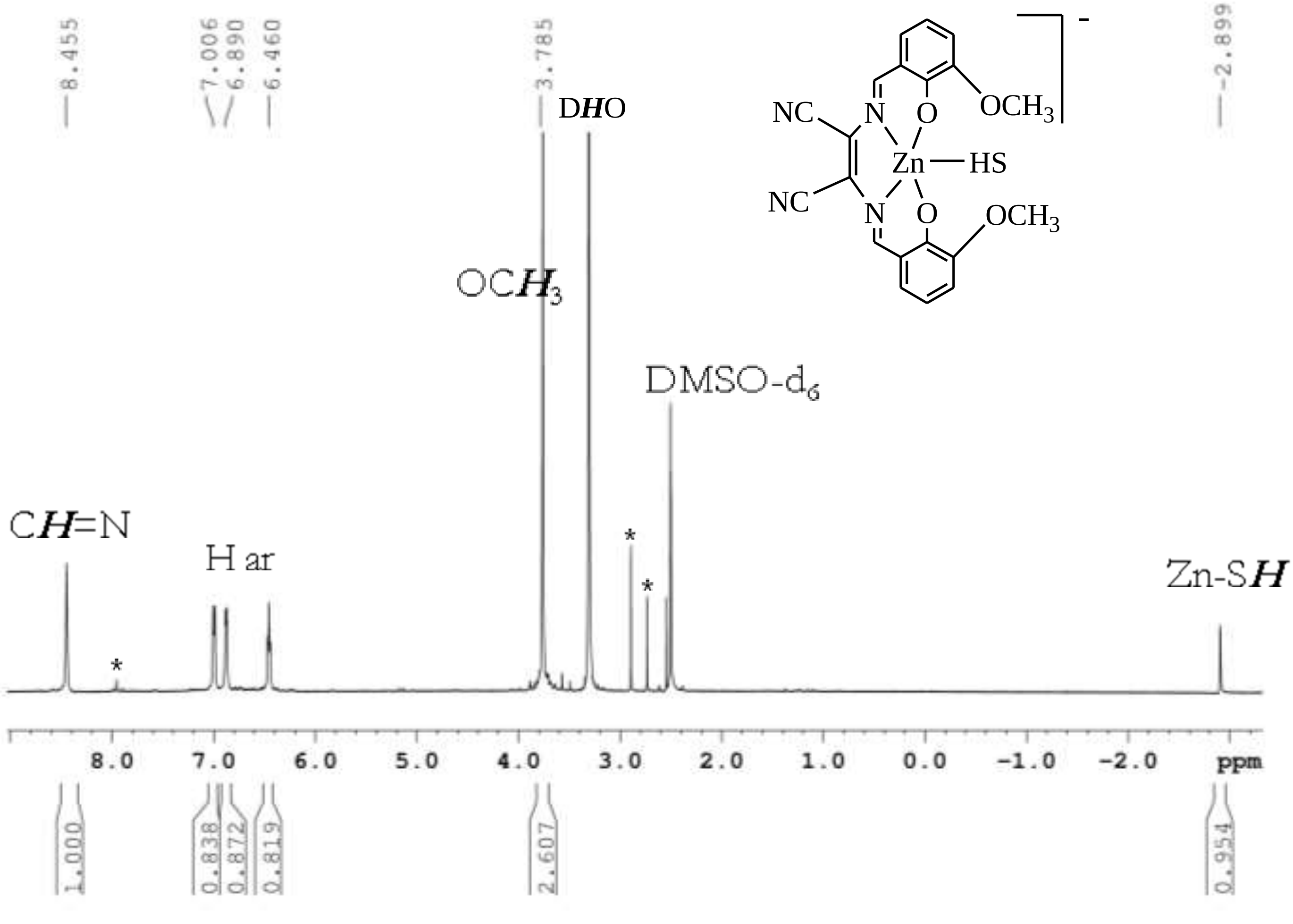


**Figure S13.** $^1$H NMR spectrum of complex **2** in DMSO-$d_6$ after the addition of an excess of $HS^-$. Peaks denoted with * correspond to DMF used for synthesis. [complex **2**] = $5\times10^{-2}$ M; [NaSH] = 0.25 M.

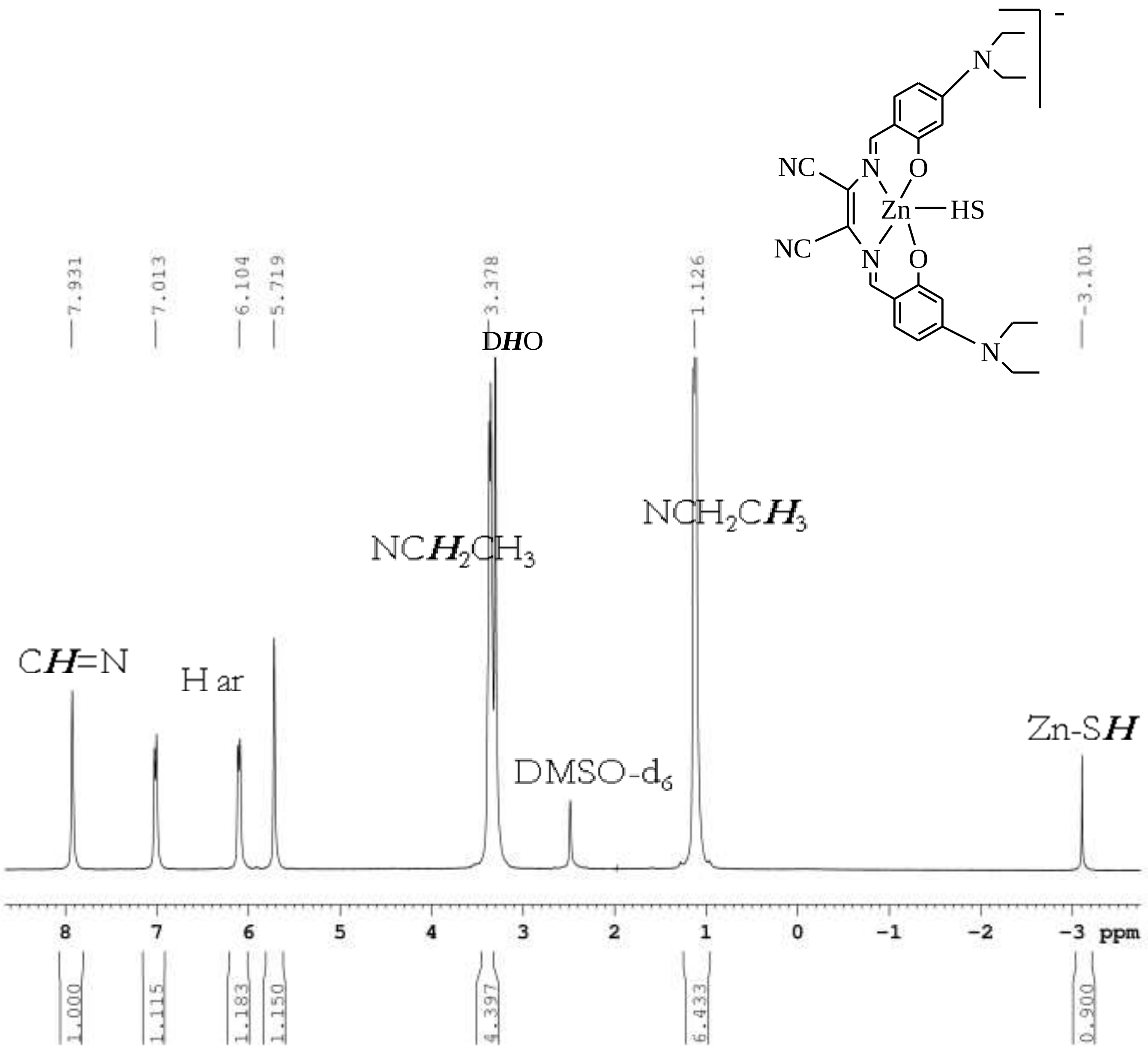


**Figure S14.** $^{1}$H NMR spectrum of complex **3** in DMSO-$d_6$ after the addition of an excess of $HS^{-}$. [complex **3**] = $5\times10^{-2}$ M; [NaSH] = 0.25 M.

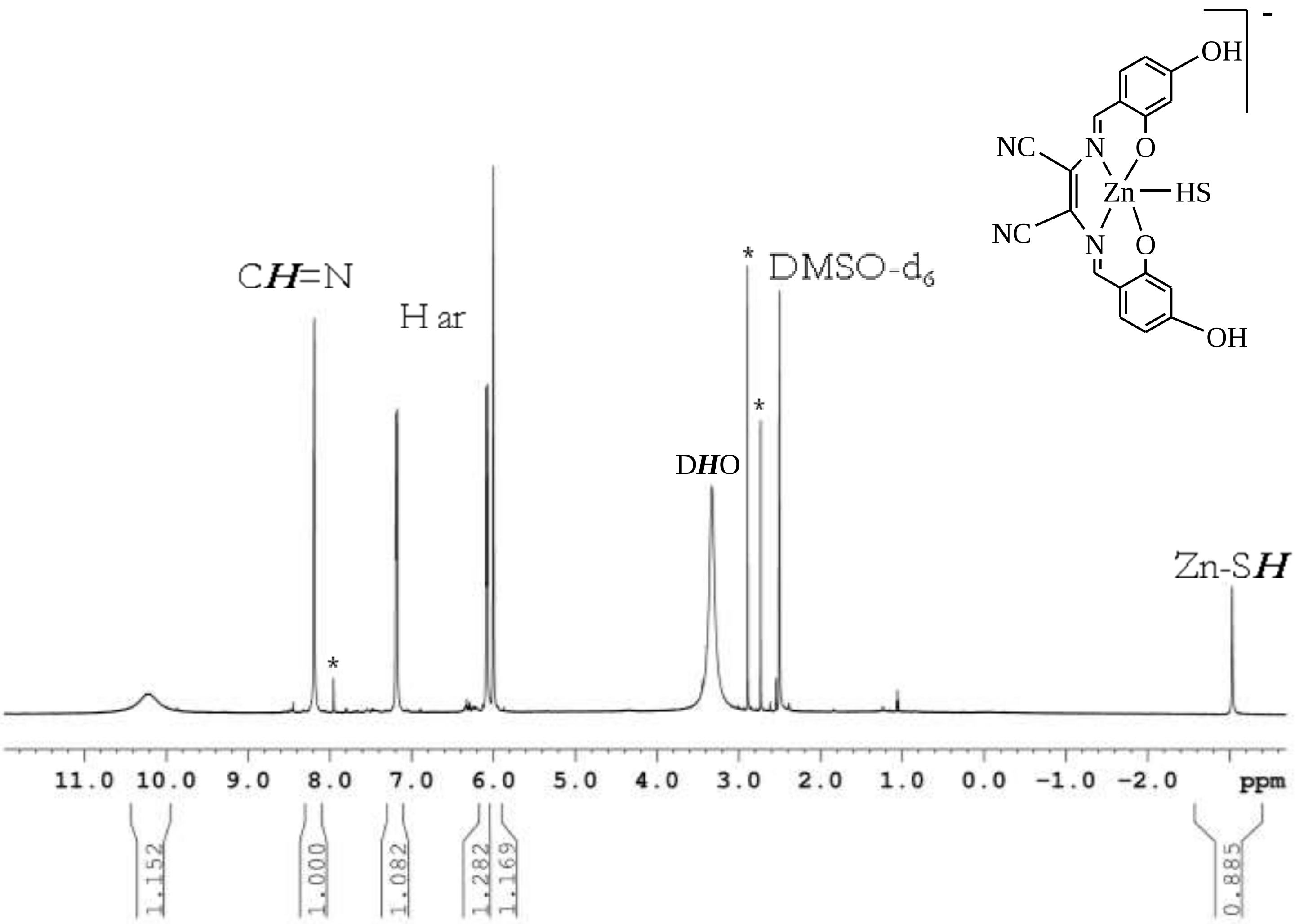


**Figure S15.** $^{1}$H NMR spectrum of complex **4** in DMSO-d6 after the addition of an excess of $HS^{-}$. Peaks denoted with * correspond to DMF used for synthesis. [complex **4**] = $5\times10^{-2}$ M; [NaSH] = 0.25 M.

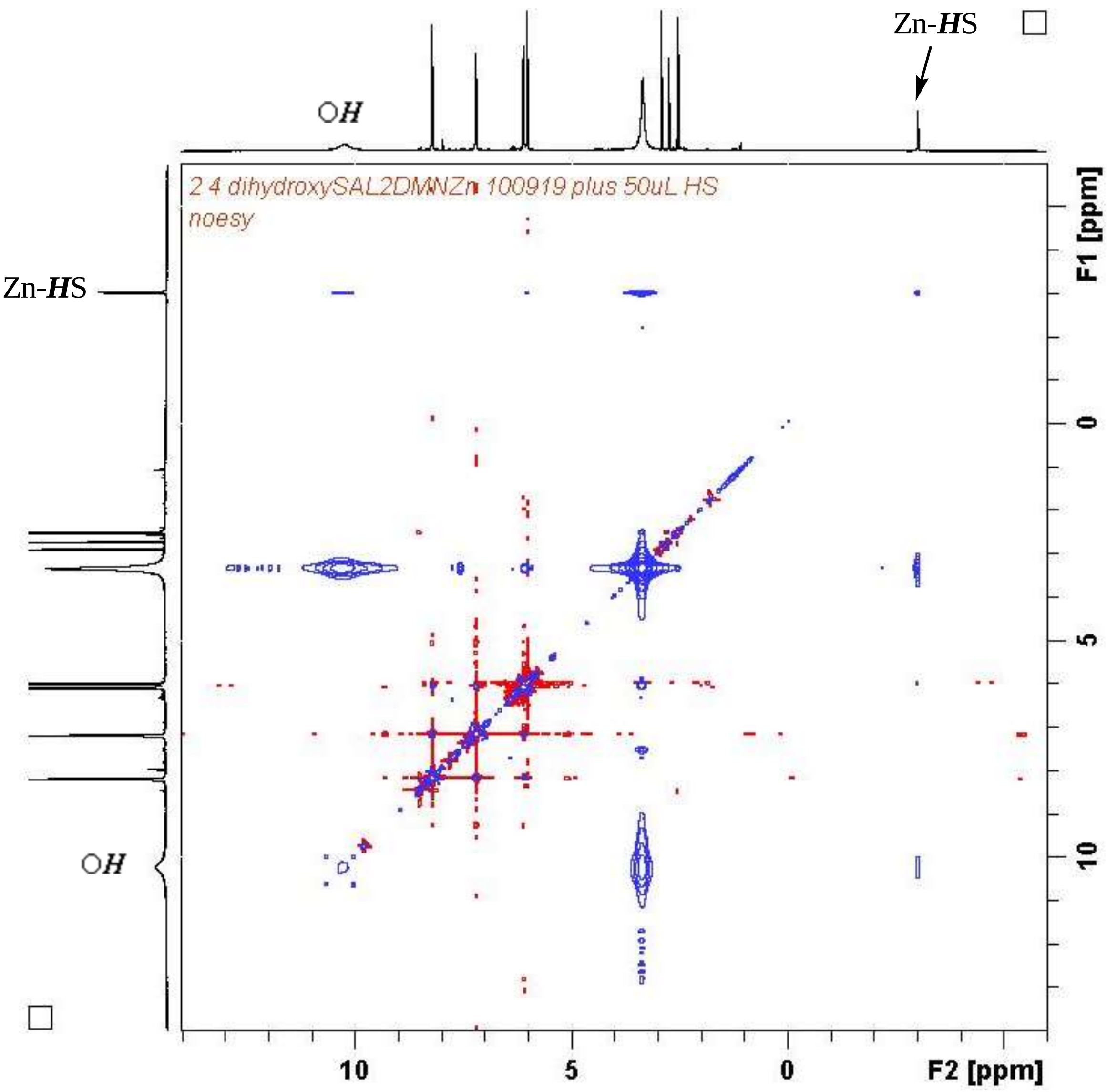


**Figure S16.** NOESY spectrum of complex **4** in DMSO-*d6* after addition of $HS^{-}$. [complex **4**] = $5\times10^{-2}$ M; [NaSH] = 0,.25 M.

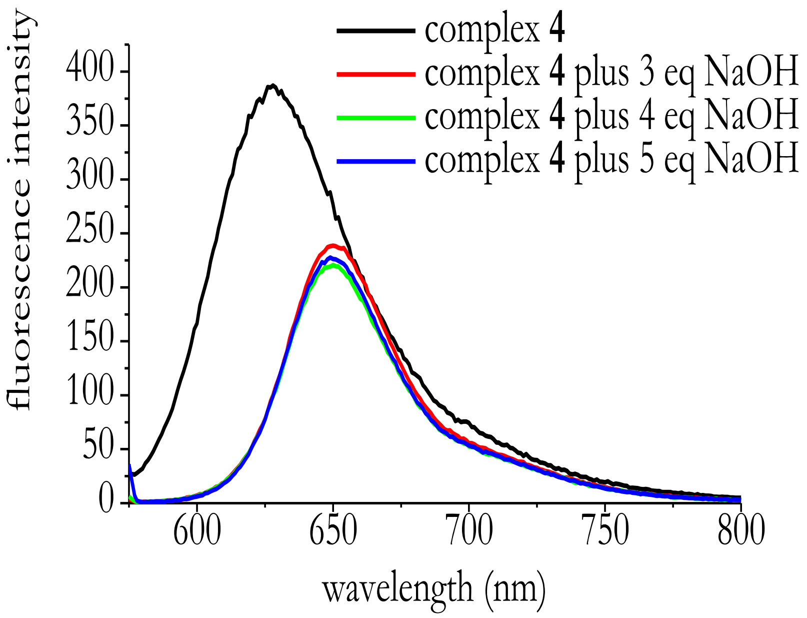


**Figure S17.** Emission spectra of complex **4** before and after the addition of NaOH in DMSO. [Complex **4**] = $1 \times 10^{-5}$ M; [NaOH] = $5 \times 10^{-5}$ M. All spectra were measured in DMSO with $\lambda_{exc}$ = 570 nm.

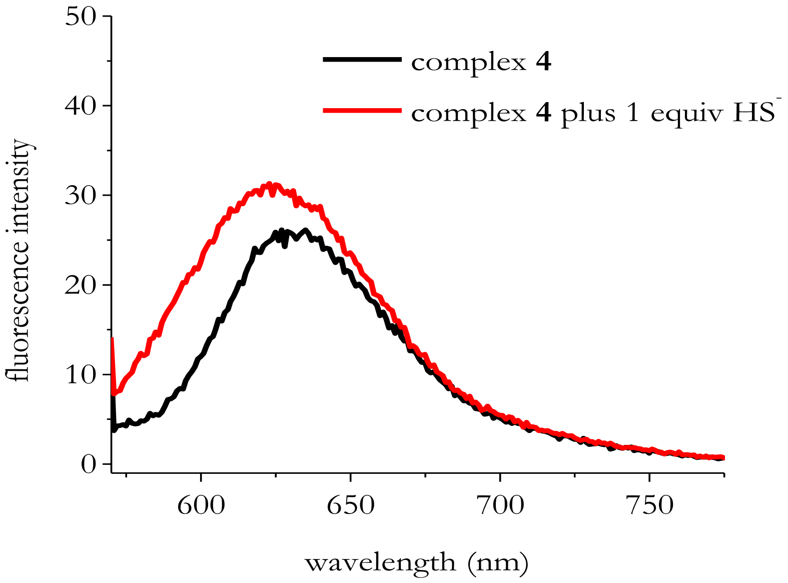


**Figure S18.** Emission spectra of complex **4** before and after the addition of 1 equiv. of NaSH. [Complex **4**] = 1 × $10^{-5}$ M; [NaSH] = 1 × $10^{-5}$M. Spectra were measured in MQ water with $\lambda_{exc}$ = 530 nm.

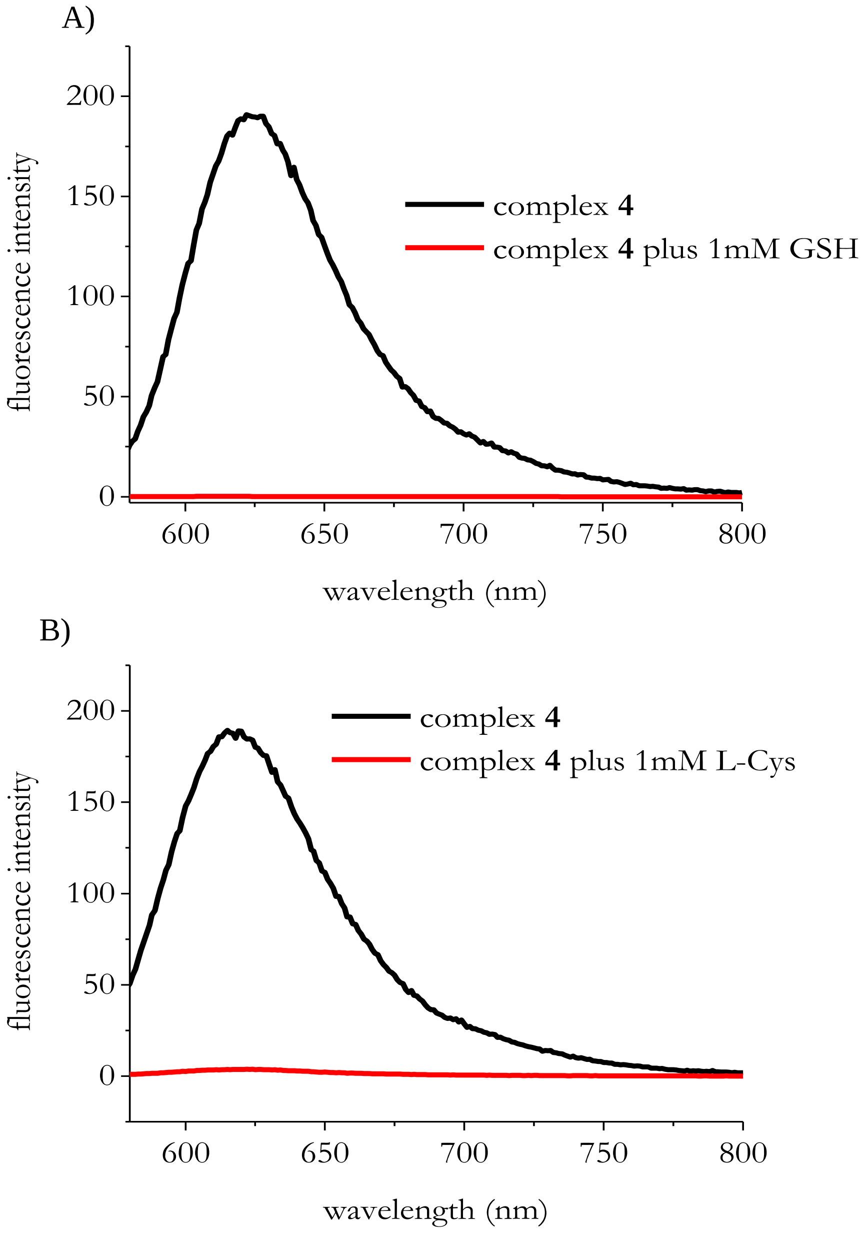


**Figure S19.** (**A**) Emission spectra of complex **4** before and after the addition of GSH in DMSO. (**B**) Emission spectra of complex **4** before and after the addition of L-cys in DMSO. [Complex **4**] = $1 \times 10^{-5}$ M; [GSH] = 1 mM; [L-Cys] = 1 mM . All spectra were measured in DMSO with $\lambda_{exc}$ = 570 nm.

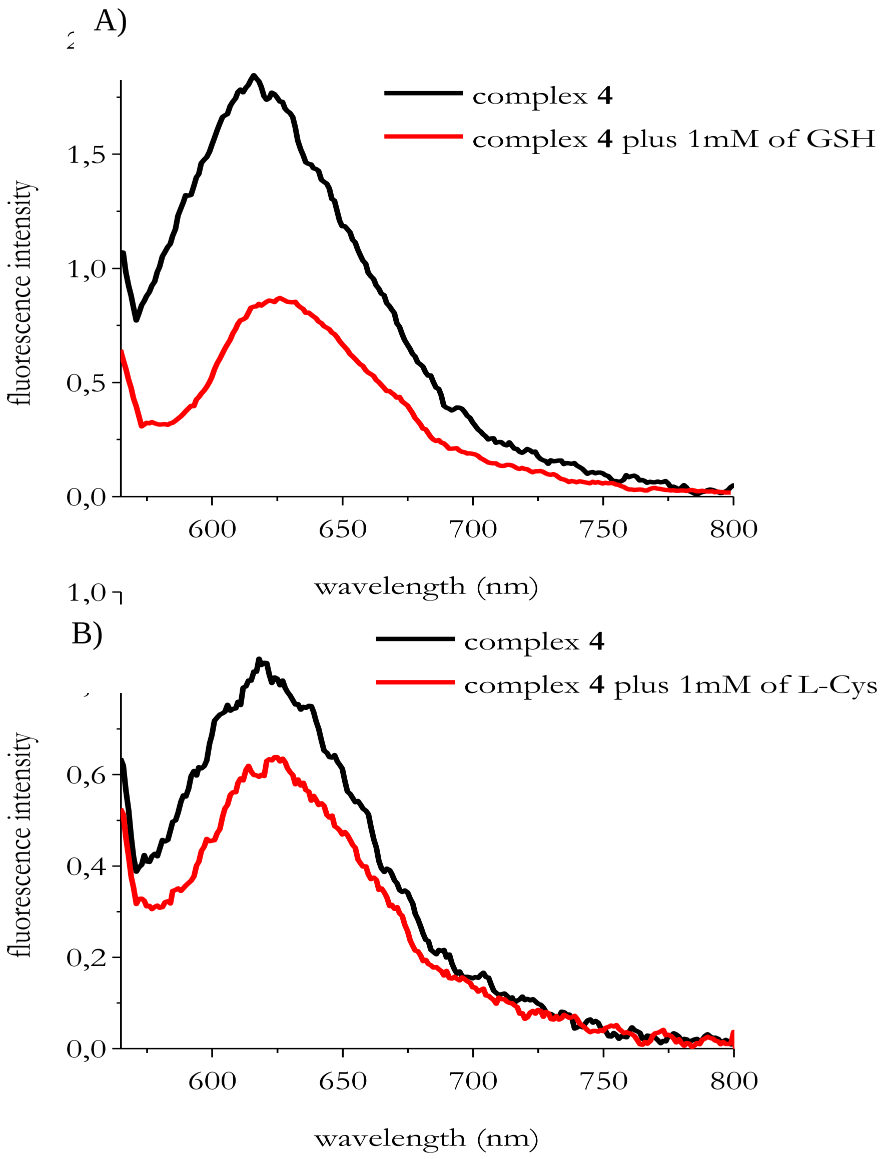


**Figure S20.** (**A**) Emission spectra of complex **4** before and after the addition of GSH in MQ water. (**B**) Emission spectra of complex **4** before and after the addition of L-cys in MQ water. [Complex **4**] = 1 × $10^{-5}$ M; [GSH] = 1 mM ; [L-Cys] = 1 mM. All spectra were measured in MQ water with $\lambda_{exc}$ = 530 nm.

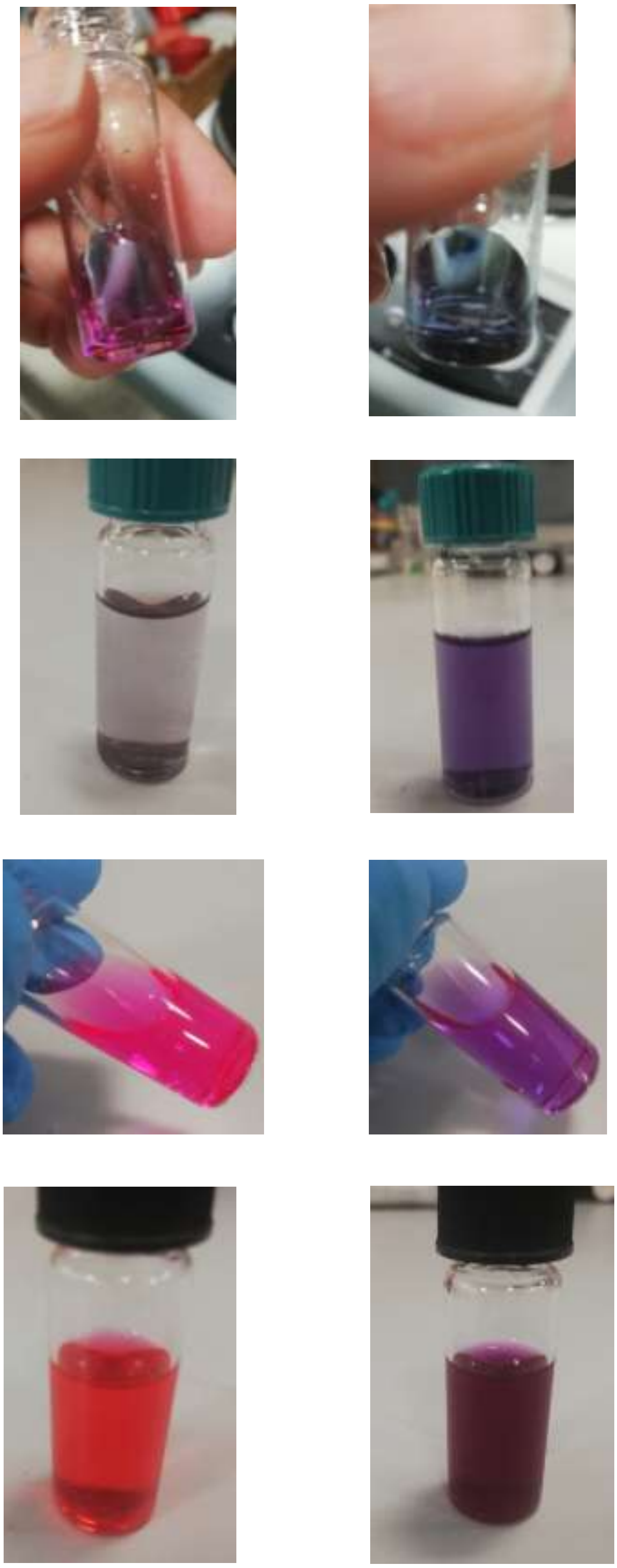

complex **1**

complex **2**

complex **3**

complex **4**

**Figure S21.** Real color images of DMSO solutions of complexes **1-4** before (left column) and after treatment with 5 equivs of $HS^{-}$ (right column).

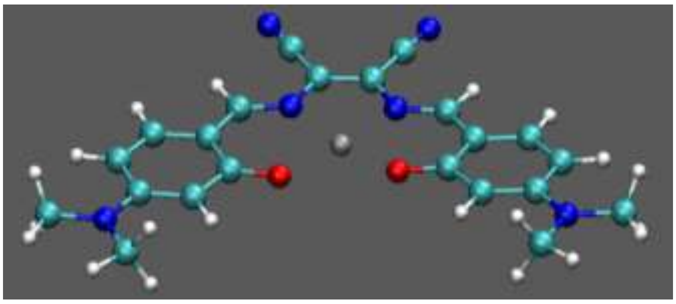

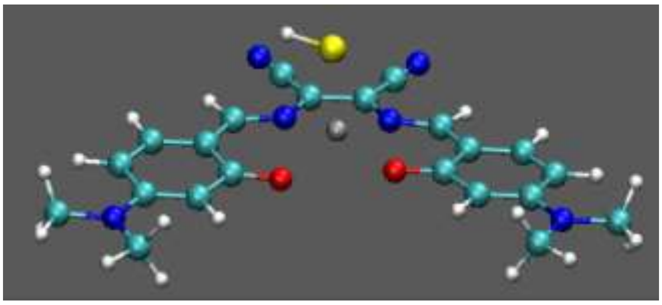

**Figure S22.** Optimized geometry for complex **3** (top) and its adduct with $HS^{-}$ (bottom).

## Fitting of experimental UV-vis spectra

We have recorded UV-vis spectra at 25 different $HS^-$ concentrations (ranging from 0.00366 mM to 0.0915 mM), with the concentration of complex **1** fixed to 0.0183 mM. Inspection of figure S23, where some experimental spectra are reported, shows that for increasing concentrations of $HS^-$, the signal at 570 nm presents a shift to longer wavelengths with a pronounced decrease in absorbance at higher HS- concentration, while the peak at 370 nm presents a progressive lowering, suggesting that three different species are forming.

The Principal Component Analysis performed with the Hyperquad package indicates that three species are present at equilibrium, i.e. complex **1** (M), and its single (ML) and double adducts ($ML_2$) with $HS^-$. Fitting of the experimental spectra (some of which are shown in fig S23 A) allowed us to evaluate the molar absorbances (reported in fig S25) and the relative abundances of ML and $ML_2$ for different concentration of $HS^-$, some of which are reported in Table S1.

The full experiment with all the 25 traces is displayed in fig S23 B.

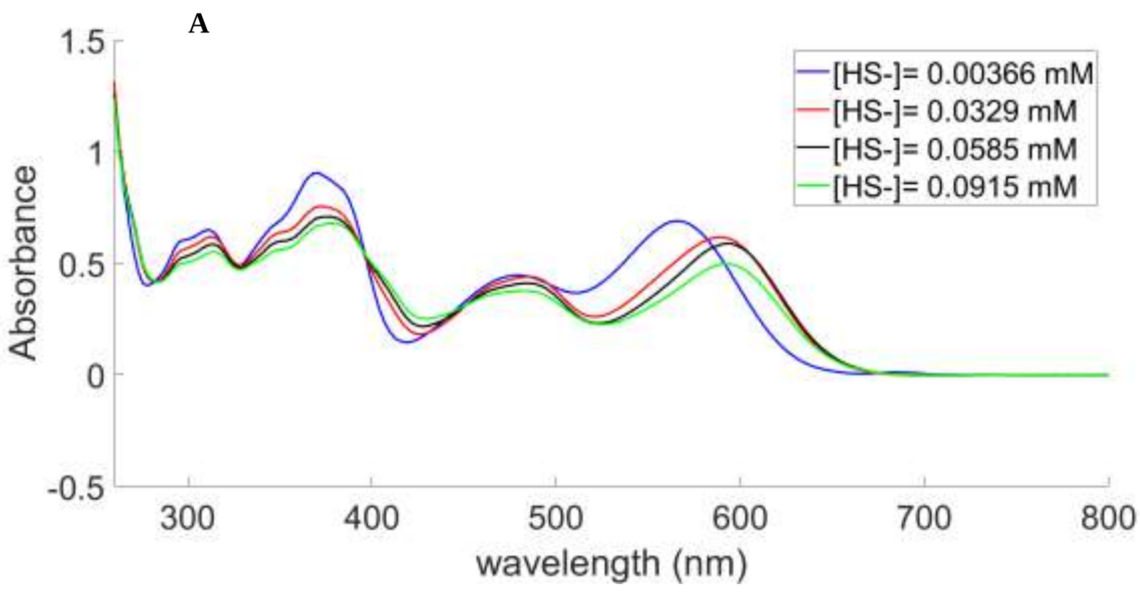


**Figure S23A.** Electronic absorption spectra of solutions of complex **1** (0.0183 mM) for different $HS^-$ concentrations.

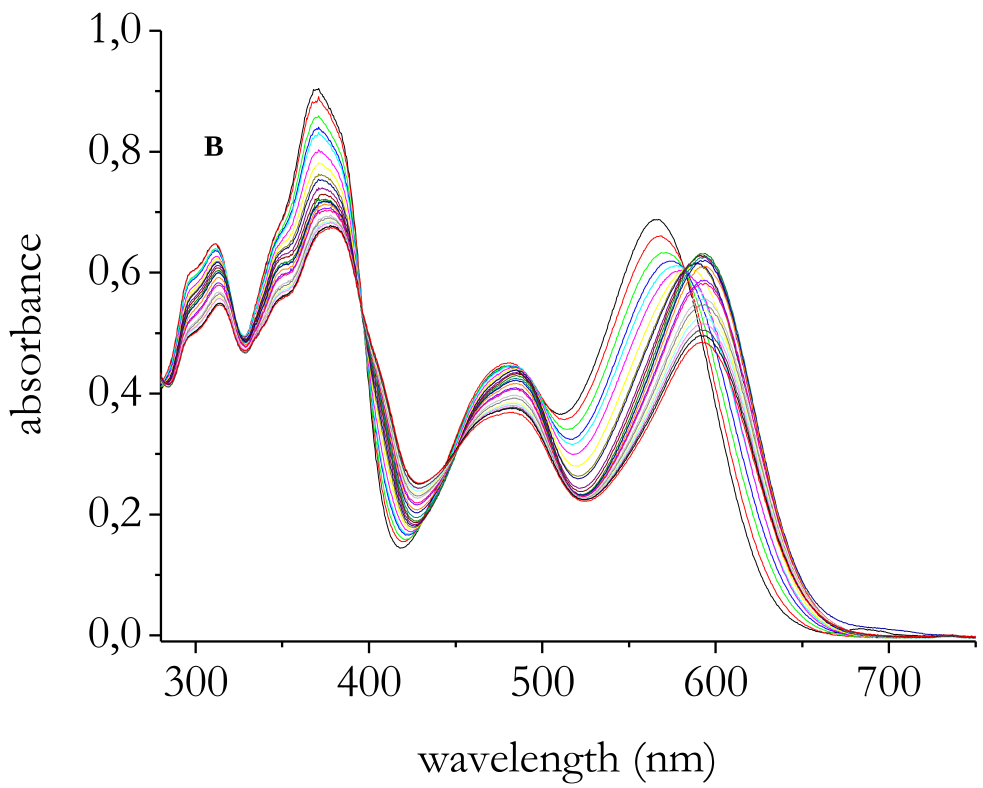


**Figure S23B.** Electronic absorption spectra of solutions of complex **1** (0.0183 mM).$HS^-$ concentration in the range 0.00366mM - 0.0915mM.

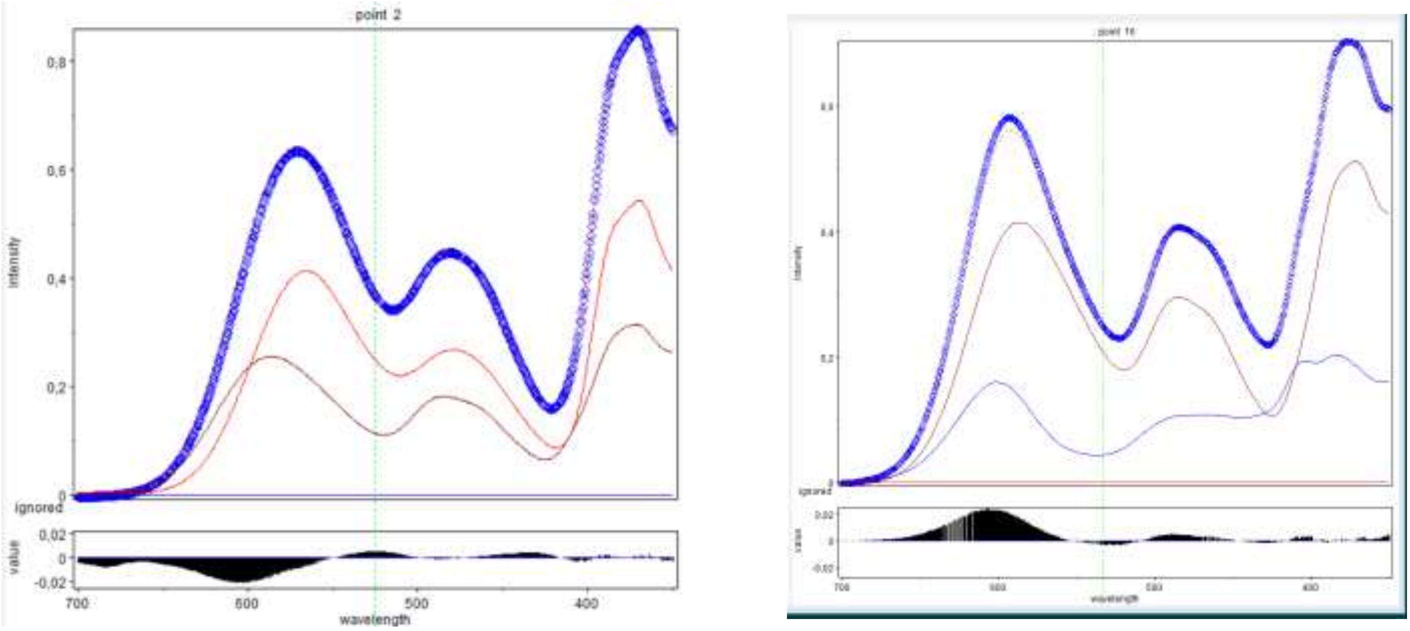


**Figure S24.** Experimental (blue diamonds) and simulated (red dashed lines) spectra of a solution of 0.0183 mM of complex **1** with the addition of 0.0256 mM (left) and 0.0585 mM (right) $HS^{-}$. Full lines are the predicted individual spectra for M (red), ML (brown) and $ML_2$ (blue). At the bottom differences between predicted and experimental spectra are displayed.

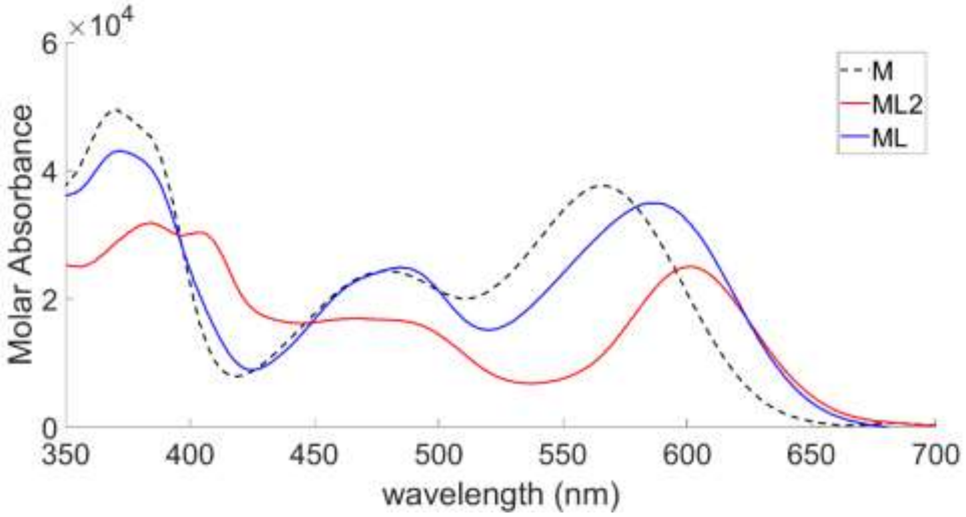


**Figure S25.** Predicted (ML, blue line; $ML_2$, red line) and experimental (M, black dashed line) molar absorbances for complex **1** (M) and its adducts with $HS^-$ (ML and $ML_2$).

| $[HS^-]$ (mM) | % M | % ML | $\%ML_2$ |
|---|---|---|---|
| **0.00732** | 60.1 | 39.8 | 0.001 |
| **0.0256** | 0.37 | 91.2 | 8.43 |
| **0.0439** | 0.08 | 74.9 | 25.0 |
| **0.0585** | 0.04 | 65.5 | 34.9 |
| **0.0841** | 0.02 | 50.9 | 49.0 |

**Table S1.** Relative abundances for complex **1** of the species M, ML and $ML_2$ for different $HS^-$ concentrations

## HOMO and LUMO of the complexes under study

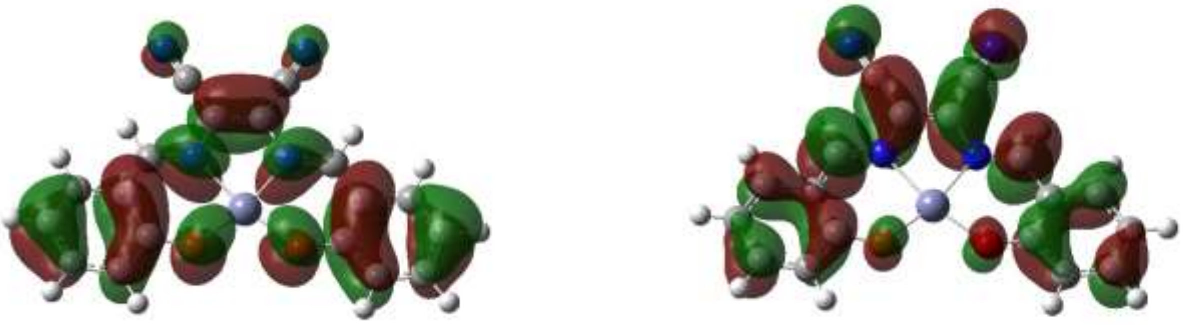

**Figure S26.** HOMO (left) and LUMO (right) of complex **1**

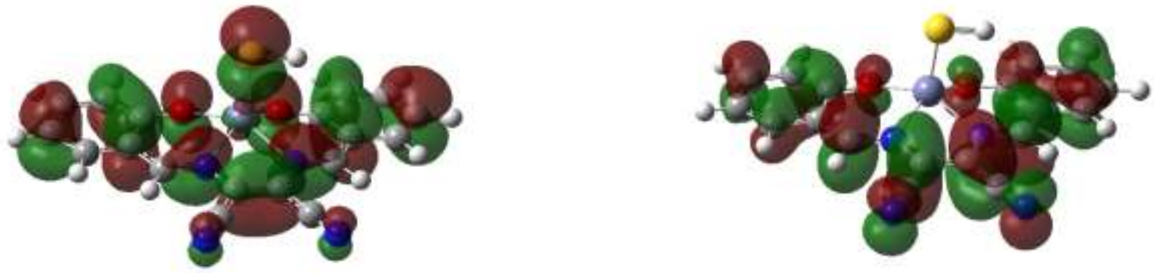

**Figure S27.** HOMO (left) and LUMO (right) of complex **1** + $HS^{-}$

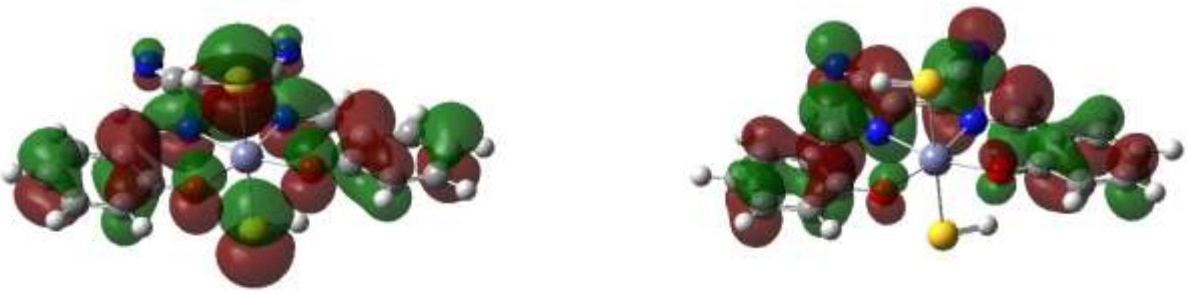

**Figure S28.** HOMO (left) and LUMO (right) of complex **1** + 2 $HS^{-}$

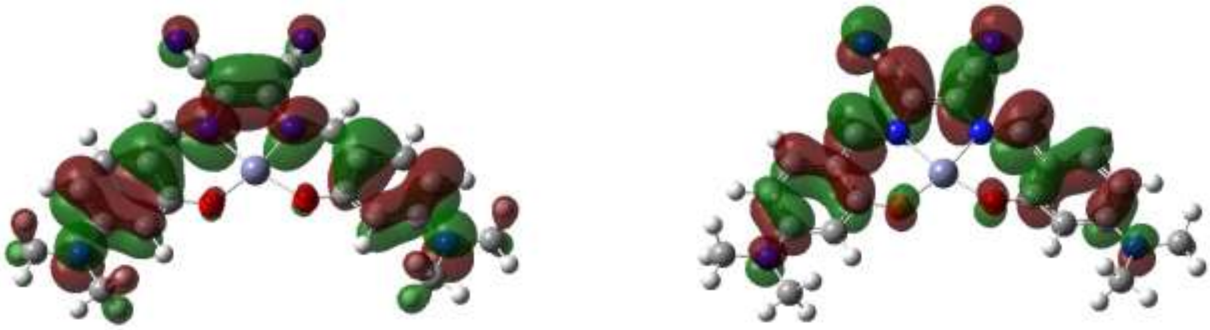

**Figure S29.** HOMO (left) and LUMO (right) of complex **3**

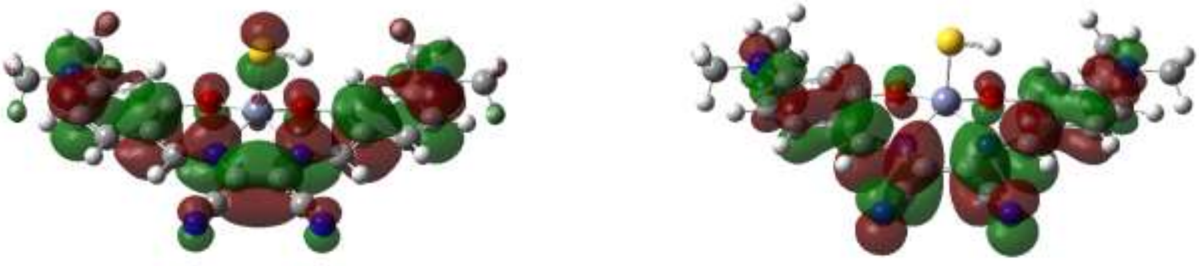

**Figure S30.** HOMO (left) and LUMO (right) of complex **3** + $HS^{-}$

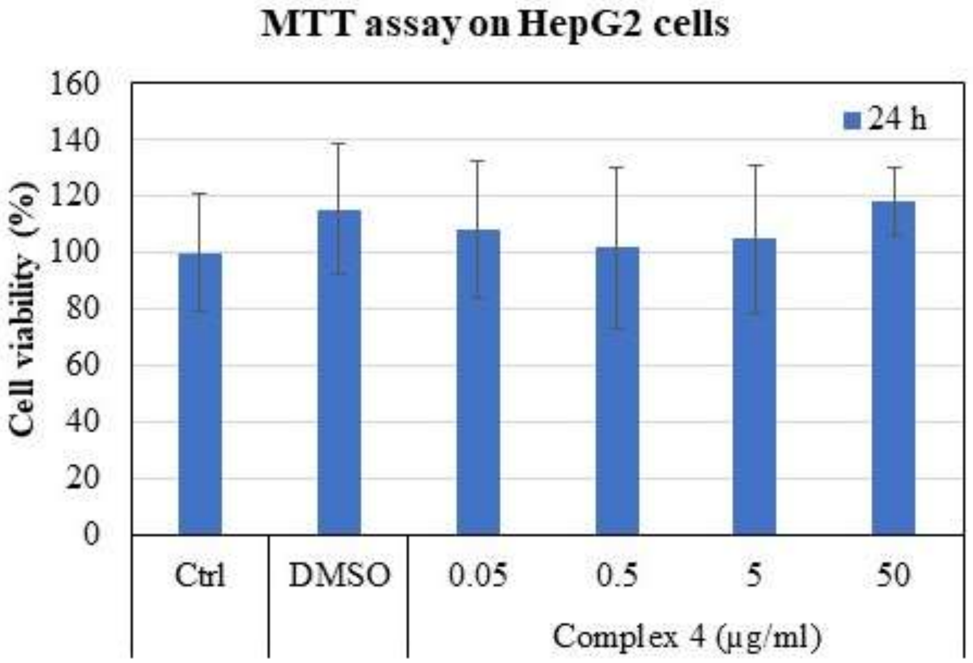


**Figure S31.** Cytotoxicity evaluation of complex **4** in HepG2 cell line by MTT assay. Cells were incubated 24 h with increasing concentrations of complex **4**. Non-treated cells (Ctrl) and cells incubated with DMSO (DMSO) were used as controls. Cell viability was compared with non-treated cells (Ctrl) and data were reported as the mean percentage ± standard deviation.

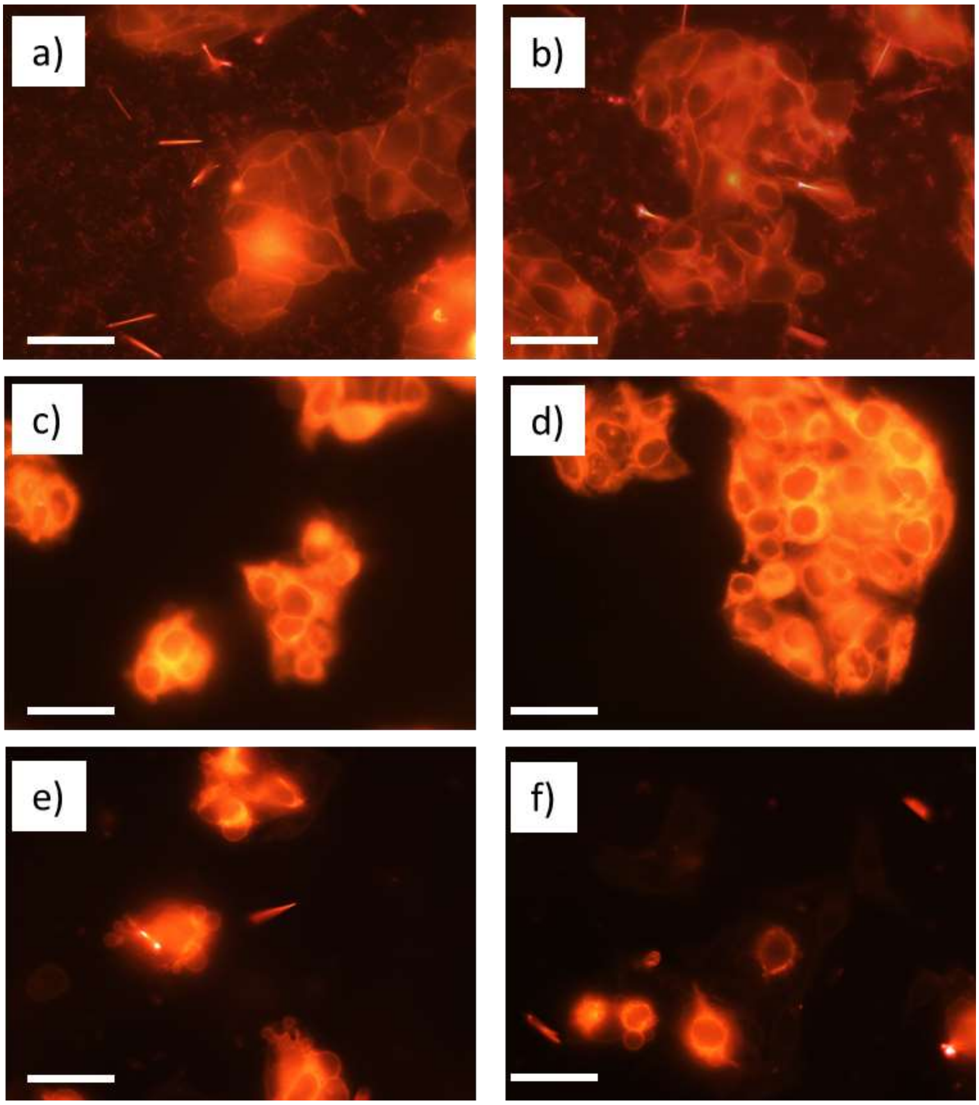


**Figure S32.** Additional fluorescence microscopy images of HepG2 cells after 10 min treatment with 120 µM complex **4** (a and b), 120 µM adduct (c and d), and 120µM complex **4** + 250 µM NaSH (e and f). Magnification bar 50 µm.

# Cartesian Coordinates of the optimized structures

Complex **1**

**$S_0$**

| | | | |
|---|---|---|---|
| N | 1.335645 | 0.983418 | 0.000600 |
| N | -1.336304 | 0.983163 | -0.010622 |
| O | 1.518794 | -1.861085 | 0.029365 |
| O | -1.518486 | -1.861168 | -0.032912 |
| C | 5.610283 | -1.274353 | -0.015535 |
| C | 2.786136 | -1.624206 | 0.015534 |
| C | 4.786113 | -0.188383 | -0.016573 |
| C | 5.034621 | -2.558594 | 0.002039 |
| C | 3.678572 | -2.727618 | 0.017564 |
| C | 3.373233 | -0.315544 | -0.001694 |
| C | 0.680965 | 2.196866 | -0.005131 |
| C | -0.681873 | 2.196723 | -0.009749 |
| C | -1.406622 | 3.428465 | -0.012987 |
| N | -2.017945 | 4.399038 | -0.015691 |
| C | -2.785884 | -1.624807 | -0.015241 |
| C | -5.610035 | -1.275999 | 0.023918 |
| C | -3.373452 | -0.316348 | -0.000483 |
| C | -3.677837 | -2.728589 | -0.010319 |
| C | -5.033905 | -2.560058 | 0.009124 |
| C | -4.786316 | -0.189688 | 0.018345 |
| C | -2.633936 | 0.889753 | -0.002210 |
| H | -3.225484 | 1.806598 | 0.005080 |
| C | 2.633335 | 0.890297 | -0.005486 |
| H | 3.224619 | 1.807289 | -0.014712 |
| H | 3.237505 | -3.715608 | 0.030697 |
| H | 5.677727 | -3.430243 | 0.003039 |
| H | 6.684585 | -1.155358 | -0.027993 |
| H | 5.207929 | 0.809822 | -0.029832 |
| H | -3.236401 | -3.716439 | -0.021347 |
| H | -5.676644 | -3.431966 | 0.013552 |
| H | -6.684341 | -1.157394 | 0.039476 |
| H | -5.208503 | 0.808386 | 0.029479 |
| C | 1.405473 | 3.428732 | -0.006959 |
| N | 2.016557 | 4.399458 | -0.008356 |
| Zn | 0.000133 | -0.645587 | -0.003191 |

**$S_1$**

| | | | |
|---|---|---|---|
| N | 1.349916 | 1.070656 | -0.046541 |
| N | -1.351762 | 1.072741 | 0.045894 |
| O | 1.476847 | -1.752099 | 0.421696 |
| O | -1.473497 | -1.754242 | -0.395135 |
| C | 5.541034 | -1.370963 | -0.103790 |
| C | 2.733051 | -1.588173 | 0.216248 |
| C | 4.768177 | -0.247285 | -0.145315 |
| C | 4.929644 | -2.628538 | 0.090647 |
| C | 3.575489 | -2.730279 | 0.232997 |
| C | 3.356998 | -0.295202 | -0.009195 |
| C | 0.714467 | 2.247849 | -0.020153 |
| C | -0.715520 | 2.248913 | 0.011559 |
| C | -1.424866 | 3.476796 | -0.008582 |
| N | -2.030078 | 4.455370 | -0.027476 |
| C | -2.731463 | -1.589263 | -0.201961 |
| C | -5.541715 | -1.371674 | 0.094928 |
| C | -3.357590 | -0.294986 | 0.010027 |
| C | -3.573226 | -2.731842 | -0.219406 |
| C | -4.928450 | -2.630046 | -0.088180 |
| C | -4.769756 | -0.247309 | 0.135861 |
| C | -2.668769 | 0.932081 | 0.077507 |
| H | -3.280713 | 1.828253 | 0.160041 |
| C | 2.666842 | 0.930617 | -0.082581 |
| H | 3.277562 | 1.826529 | -0.176012 |
| H | 3.095609 | -3.687064 | 0.390744 |
| H | 5.541857 | -3.520864 | 0.123154 |
| H | 6.614277 | -1.301851 | -0.216108 |
| H | 5.231168 | 0.719857 | -0.299503 |
| H | -3.091524 | -3.689182 | -0.368040 |
| H | -5.540009 | -3.522821 | -0.120834 |
| H | -6.615797 | -1.302542 | 0.198880 |
| H | -5.234538 | 0.720398 | 0.280773 |
| C | 1.424697 | 3.475288 | -0.009466 |
| N | 2.030003 | 4.453924 | 0.002501 |
| Zn | -0.000110 | -0.534069 | 0.015608 |

Complex **1 + $HS^-$**

**$S_0$**

| | | | |
|---|---|---|---|
| N | 1.367065 | 0.978071 | 0.067630 |
| N | -1.302757 | 0.989571 | 0.101968 |
| O | 1.531122 | -1.844126 | 0.070446 |
| O | -1.437634 | -1.816164 | -0.041525 |
| C | 5.621772 | -1.301566 | -0.171913 |
| C | 2.793344 | -1.628305 | -0.000433 |

| | | | |
|---|---|---|---|
| C | 4.805042 | -0.209108 | -0.122341 |
| C | 5.035979 | -2.581234 | -0.144136 |
| C | 3.680850 | -2.739256 | -0.066025 |
| C | 3.396108 | -0.324546 | -0.034011 |
| C | 0.716316 | 2.190152 | 0.021737 |
| C | -0.646950 | 2.196301 | 0.043741 |
| C | -1.365447 | 3.432525 | -0.013865 |
| N | -1.977646 | 4.401923 | -0.058934 |
| C | -2.704569 | -1.616017 | -0.018114 |
| C | -5.542075 | -1.319876 | 0.014720 |
| C | -3.322973 | -0.320597 | 0.056785 |
| C | -3.583227 | -2.733739 | -0.086930 |
| C | -4.942261 | -2.590762 | -0.065072 |
| C | -4.735378 | -0.219896 | 0.066111 |
| C | -2.595375 | 0.897083 | 0.063096 |
| H | -3.196260 | 1.808465 | 0.019298 |
| C | 2.657382 | 0.886017 | -0.030428 |
| H | 3.250065 | 1.798369 | -0.128274 |
| H | 3.233406 | -3.724587 | -0.043631 |
| H | 5.671044 | -3.458210 | -0.185102 |
| H | 6.695234 | -1.190307 | -0.235870 |
| H | 5.233921 | 0.786267 | -0.150619 |
| H | -3.125371 | -3.712694 | -0.146845 |
| H | -5.569506 | -3.473174 | -0.110002 |
| H | -6.618541 | -1.220619 | 0.028697 |
| H | -5.175388 | 0.769746 | 0.116978 |
| C | 1.440621 | 3.420900 | -0.066724 |
| N | 2.054560 | 4.387590 | -0.138677 |
| Zn | 0.038321 | -0.648729 | 0.663487 |
| S | -0.017765 | -0.557587 | 3.008042 |
| H | -1.333081 | -0.283623 | 3.088813 |

**$S_1$**

| | | | |
|---|---|---|---|
| N | 1.375055 | 1.024629 | 0.043218 |
| N | -1.322933 | 1.049986 | 0.080590 |
| O | 1.527391 | -1.824136 | 0.120608 |
| O | -1.422012 | -1.757616 | -0.161274 |
| C | 5.603747 | -1.353425 | -0.177578 |
| C | 2.785818 | -1.634590 | 0.029314 |
| C | 4.811429 | -0.241580 | -0.145125 |
| C | 5.007934 | -2.631170 | -0.111212 |
| C | 3.652488 | -2.763174 | -0.013612 |
| C | 3.399459 | -0.320051 | -0.043665 |

| | | | |
|---|---|---|---|
| C | 0.746893 | 2.209149 | 0.022021 |
| C | -0.680674 | 2.222750 | 0.054742 |
| C | -1.380865 | 3.456528 | 0.042056 |
| N | -1.984138 | 4.437412 | 0.029734 |
| C | -2.686517 | -1.602840 | -0.078850 |
| C | -5.514380 | -1.385196 | 0.051795 |
| C | -3.330023 | -0.305076 | 0.050355 |
| C | -3.531004 | -2.746972 | -0.133633 |
| C | -4.891608 | -2.645534 | -0.071938 |
| C | -4.744843 | -0.257605 | 0.105759 |
| C | -2.638047 | 0.923940 | 0.080382 |
| H | -3.251842 | 1.824316 | 0.089375 |
| C | 2.687499 | 0.897429 | -0.046962 |
| H | 3.289734 | 1.799967 | -0.145928 |
| H | 3.183975 | -3.737272 | 0.040376 |
| H | 5.633688 | -3.514615 | -0.137479 |
| H | 6.678312 | -1.258894 | -0.255056 |
| H | 5.262775 | 0.742102 | -0.199890 |
| H | -3.041589 | -3.707717 | -0.227235 |
| H | -5.500321 | -3.540112 | -0.115504 |
| H | -6.592584 | -1.315486 | 0.100255 |
| H | -5.217848 | 0.713146 | 0.196885 |
| C | 1.463323 | 3.430800 | -0.046692 |
| N | 2.074754 | 4.405261 | -0.103008 |
| Zn | 0.029453 | -0.574638 | 0.582172 |
| S | -0.113271 | -0.572300 | 2.910508 |
| H | -1.339281 | -0.019162 | 2.956572 |

Complex **1 + 2HS$^{-}$**

**$S_0$**

| | | | |
|---|---|---|---|
| N | 1.354940 | 0.894652 | 0.030555 |
| N | -1.363217 | 0.897341 | -0.053332 |
| O | 1.668106 | -1.979545 | 0.187046 |
| O | -1.683935 | -1.977097 | -0.188248 |
| C | 5.716393 | -1.203926 | -0.116592 |
| C | 2.903966 | -1.686551 | 0.085443 |
| C | 4.834366 | -0.162202 | -0.111188 |
| C | 5.207117 | -2.513593 | -0.019792 |
| C | 3.865288 | -2.744209 | 0.077547 |
| C | 3.432858 | -0.349202 | -0.015992 |
| C | 0.679796 | 2.089940 | 0.000578 |
| C | -0.685227 | 2.091314 | -0.038830 |
| C | -1.389303 | 3.339243 | -0.050457 |
| N | -1.991152 | 4.316527 | -0.061679 |
| C | -2.918294 | -1.680594 | -0.078626 |
| C | -5.728095 | -1.190022 | 0.140728 |
| C | -3.443619 | -0.341239 | 0.014742 |
| C | -3.881701 | -2.736053 | -0.052685 |

| | | | |
|---|---|---|---|
| C | -5.222251 | -2.501657 | 0.052932 |
| C | -4.843995 | -0.150293 | 0.118389 |
| C | -2.655120 | 0.840815 | 0.005179 |
| H | -3.224584 | 1.772341 | 0.058551 |
| C | 2.646911 | 0.834603 | -0.022387 |
| H | 3.218739 | 1.764252 | -0.082803 |
| H | 3.476517 | -3.752003 | 0.151816 |
| H | 5.892175 | -3.353465 | -0.022588 |
| H | 6.781019 | -1.031127 | -0.192489 |
| H | 5.205963 | 0.854121 | -0.183629 |
| H | -3.495583 | -3.745351 | -0.119996 |
| H | -5.908977 | -3.340006 | 0.069551 |
| H | -6.791752 | -1.014223 | 0.223057 |
| H | -5.212892 | 0.867485 | 0.183885 |
| C | 1.386795 | 3.336279 | -0.003814 |
| N | 1.990887 | 4.312241 | -0.005179 |
| Zn | -0.006358 | -0.818177 | -0.004465 |
| S | -0.253247 | -0.824751 | 2.591048 |
| H | -1.566239 | -0.528497 | 2.575008 |
| S | 0.240071 | -0.838828 | -2.599645 |
| H | 1.554566 | -0.548990 | -2.585986 |

**$S_1$**

| | | | |
|---|---|---|---|
| N | 1.366917 | 0.960436 | 0.006018 |
| N | -1.374771 | 0.963330 | -0.024256 |
| O | 1.630012 | -1.925353 | 0.141958 |
| O | -1.644321 | -1.923761 | -0.127762 |
| C | 5.685941 | -1.274648 | -0.092229 |
| C | 2.874688 | -1.675933 | 0.061470 |
| C | 4.840337 | -0.200220 | -0.090329 |
| C | 5.148425 | -2.575963 | -0.018771 |
| C | 3.797882 | -2.764252 | 0.053833 |
| C | 3.431653 | -0.335857 | -0.018419 |
| C | 0.710594 | 2.130592 | -0.016184 |
| C | -0.715861 | 2.132228 | -0.017949 |
| C | -1.411435 | 3.368863 | -0.000576 |
| N | -2.012548 | 4.352062 | 0.011809 |
| C | -2.888300 | -1.670410 | -0.048270 |
| C | -5.698521 | -1.260879 | 0.104506 |
| C | -3.442175 | -0.328207 | 0.016647 |
| C | -3.814035 | -2.756391 | -0.026516 |
| C | -5.164044 | -2.564186 | 0.045808 |
| C | -4.850466 | -0.188519 | 0.088592 |
| C | -2.687556 | 0.869237 | 0.012193 |
| H | -3.272991 | 1.788616 | 0.049523 |
| C | 2.679536 | 0.863110 | -0.029493 |

| | | | |
|---|---|---|---|
| H | 3.266899 | 1.780693 | -0.078723 |
| H | 3.371876 | -3.757932 | 0.110819 |
| H | 5.812556 | -3.431614 | -0.020943 |
| H | 6.756326 | -1.129585 | -0.149703 |
| H | 5.249875 | 0.801808 | -0.147690 |
| H | -3.390393 | -3.751655 | -0.072429 |
| H | -5.830098 | -3.418239 | 0.059141 |
| H | -6.768489 | -1.112664 | 0.161707 |
| H | -5.257634 | 0.815067 | 0.134499 |
| C | 1.408941 | 3.365325 | -0.050531 |
| N | 2.012277 | 4.346906 | -0.076055 |
| Zn | -0.005991 | -0.713740 | 0.001421 |
| S | -0.197521 | -0.776528 | 2.582762 |
| H | -1.512482 | -0.490259 | 2.577923 |
| S | 0.185338 | -0.798615 | -2.580302 |
| H | 1.500492 | -0.513309 | -2.577846 |

Complex **3**

**$S_0$**

| | | | |
|---|---|---|---|
| N | 1.336187 | 1.076557 | -0.066848 |
| N | -1.326928 | 1.125771 | -0.039500 |
| O | 1.455609 | -1.759542 | 0.235863 |
| O | -1.545418 | -1.717754 | 0.033551 |
| C | 5.559263 | -1.277186 | 0.146657 |
| C | 2.734518 | -1.564428 | 0.201041 |
| C | 4.758692 | -0.189760 | 0.035748 |
| C | 4.974895 | -2.575768 | 0.293079 |
| C | 3.582256 | -2.674801 | 0.316343 |
| C | 3.339843 | -0.261752 | 0.052827 |
| C | 0.709581 | 2.298045 | -0.135736 |
| C | -0.657730 | 2.323576 | -0.114880 |
| C | -1.360334 | 3.565183 | -0.174519 |
| N | -1.957403 | 4.544540 | -0.220084 |
| C | -2.816375 | -1.476999 | 0.031913 |
| C | -5.629348 | -1.090358 | 0.026931 |
| C | -3.376567 | -0.146492 | 0.000409 |
| C | -3.701380 | -2.563955 | 0.059588 |
| C | -5.089628 | -2.415976 | 0.059248 |
| C | -4.792244 | -0.025276 | -0.002404 |
| C | -2.634742 | 1.039369 | -0.043851 |
| H | -3.215887 | 1.962174 | -0.088684 |
| C | 2.640117 | 0.945205 | -0.056256 |
| H | 3.253195 | 1.844798 | -0.133681 |
| H | 3.092323 | -3.629582 | 0.425422 |
| H | 6.629941 | -1.153911 | 0.125686 |

| | | | |
|---|---|---|---|
| H | 5.215669 | 0.787325 | -0.072243 |
| H | -3.244046 | -3.540624 | 0.082563 |
| H | -6.695267 | -0.929539 | 0.023956 |
| H | -5.215933 | 0.972182 | -0.028477 |
| C | 1.456015 | 3.512530 | -0.214574 |
| N | 2.087437 | 4.469142 | -0.277350 |
| Zn | -0.019299 | -0.502115 | 0.052094 |
| N | 5.764877 | -3.669901 | 0.405171 |
| C | 7.213495 | -3.551124 | 0.381502 |
| C | 5.168276 | -4.983816 | 0.557063 |
| H | 7.649404 | -4.539751 | 0.482751 |
| H | 7.566951 | -3.121795 | -0.558318 |
| H | 7.580800 | -2.934921 | 1.204956 |
| H | 5.955271 | -5.727481 | 0.629421 |
| H | 4.558186 | -5.042556 | 1.461801 |
| H | 4.537318 | -5.236280 | -0.298620 |
| N | -5.916305 | -3.488278 | 0.088306 |
| C | -5.364655 | -4.829908 | 0.121367 |
| C | -7.360031 | -3.318978 | 0.091900 |
| H | -6.176540 | -5.549729 | 0.139586 |
| H | -4.750400 | -5.030111 | -0.759932 |
| H | -4.749127 | -4.986320 | 1.010684 |
| H | -7.828849 | -4.297363 | 0.118382 |
| H | -7.697864 | -2.759549 | 0.966867 |
| H | -7.707353 | -2.803182 | -0.805814 |

**$S_1$**

| | | | |
|---|---|---|---|
| N | 1.356027 | 1.141532 | -0.091148 |
| N | -1.345338 | 1.194416 | -0.040762 |
| O | 1.441403 | -1.715117 | 0.230141 |
| O | -1.531091 | -1.673344 | -0.043645 |
| C | 5.550933 | -1.301980 | 0.117202 |
| C | 2.724418 | -1.546386 | 0.187733 |
| C | 4.769383 | -0.200574 | -0.007340 |
| C | 4.948103 | -2.589804 | 0.288726 |
| C | 3.550882 | -2.663849 | 0.313157 |
| C | 3.347690 | -0.243571 | 0.019111 |
| C | 0.744898 | 2.330653 | -0.145452 |
| C | -0.691521 | 2.358840 | -0.118006 |
| C | -1.377770 | 3.594959 | -0.177758 |
| N | -1.967160 | 4.584142 | -0.225831 |
| C | -2.806407 | -1.457323 | -0.000817 |
| C | -5.621736 | -1.110519 | 0.077892 |
| C | -3.384235 | -0.123107 | 0.017352 |
| C | -3.670167 | -2.553265 | 0.026320 |
| C | -5.063426 | -2.429539 | 0.062370 |
| C | -4.803286 | -0.029153 | 0.053973 |
| C | -2.668631 | 1.081572 | -0.014120 |
| H | -3.259974 | 1.995762 | -0.023966 |

| | | | |
|---|---|---|---|
| C | 2.674576 | 0.979987 | -0.098230 |
| H | 3.297524 | 1.868155 | -0.190171 |
| H | 3.045449 | -3.608387 | 0.439760 |
| H | 6.623576 | -1.196055 | 0.088978 |
| H | 5.243675 | 0.765600 | -0.132979 |
| H | -3.197309 | -3.522728 | 0.011438 |
| H | -6.689867 | -0.965819 | 0.106372 |
| H | -5.244288 | 0.960571 | 0.065383 |
| C | 1.476172 | 3.539923 | -0.215984 |
| N | 2.100745 | 4.506909 | -0.271289 |
| Zn | -0.018307 | -0.451026 | 0.018813 |
| N | 5.719943 | -3.692189 | 0.422206 |
| C | 7.171099 | -3.596962 | 0.413403 |
| C | 5.103279 | -4.993813 | 0.601540 |
| H | 7.589634 | -4.590335 | 0.536009 |
| H | 7.539656 | -3.188640 | -0.529656 |
| H | 7.536422 | -2.972085 | 1.231024 |
| H | 5.878427 | -5.748652 | 0.681657 |
| H | 4.497968 | -5.024239 | 1.510869 |
| H | 4.462863 | -5.248147 | -0.246119 |
| N | -5.872024 | -3.513354 | 0.082518 |
| C | -5.300327 | -4.847356 | 0.066610 |
| C | -7.318757 | -3.369351 | 0.114981 |
| H | -6.100766 | -5.579338 | 0.088879 |
| H | -4.707301 | -5.013856 | -0.835884 |
| H | -4.658434 | -5.013613 | 0.934973 |
| H | -7.770077 | -4.355925 | 0.124501 |
| H | -7.648237 | -2.837558 | 1.009884 |
| H | -7.687846 | -2.836526 | -0.763711 |

Complex **3 + HS⁻**

**$S_0$**

| | | | |
|---|---|---|---|
| N | 1.358524 | 1.090512 | 0.063473 |
| N | -1.304519 | 1.152996 | 0.023155 |
| O | 1.466317 | -1.722298 | 0.389716 |
| O | -1.491273 | -1.652391 | 0.168244 |
| C | 5.565002 | -1.299760 | 0.102905 |
| C | 2.737403 | -1.550891 | 0.300427 |
| C | 4.770583 | -0.200965 | 0.043729 |
| C | 4.971419 | -2.592591 | 0.256551 |
| C | 3.582921 | -2.675998 | 0.348472 |
| C | 3.356818 | -0.255781 | 0.136574 |
| C | 0.739452 | 2.301672 | -0.120344 |
| C | -0.628022 | 2.334393 | -0.137335 |
| C | -1.318188 | 3.570042 | -0.339255 |
| N | -1.910217 | 4.540830 | -0.499141 |
| C | -2.759290 | -1.441978 | 0.120797 |
| C | -5.581721 | -1.099023 | -0.000323 |
| C | -3.343909 | -0.122260 | 0.045579 |
| C | -3.636669 | -2.543252 | 0.123257 |

| | | | |
|---|---|---|---|
| C | -5.023880 | -2.414757 | 0.069392 |
| C | -4.756344 | -0.021553 | -0.017304 |
| C | -2.605120 | 1.068575 | -0.042301 |
| H | -3.188700 | 1.981016 | -0.188578 |
| C | 2.655475 | 0.952875 | 0.002841 |
| H | 3.271011 | 1.837846 | -0.176175 |
| H | 3.087889 | -3.627051 | 0.468717 |
| H | 6.634561 | -1.188882 | 0.025824 |
| H | 5.233566 | 0.771597 | -0.082308 |
| H | -3.168311 | -3.513641 | 0.179223 |
| H | -6.648891 | -0.953698 | -0.048006 |
| H | -5.192400 | 0.969362 | -0.080968 |
| C | 1.489934 | 3.503644 | -0.308549 |
| N | 2.126858 | 4.447512 | -0.456776 |
| Zn | -0.018627 | -0.420979 | 0.779096 |
| N | 5.755091 | -3.700888 | 0.309161 |
| C | 7.200562 | -3.597661 | 0.212702 |
| C | 5.148808 | -5.010744 | 0.444817 |
| H | 7.631023 | -4.591003 | 0.291238 |
| H | 7.513754 | -3.169352 | -0.742509 |
| H | 7.616118 | -2.987451 | 1.017634 |
| H | 5.927905 | -5.766431 | 0.457768 |
| H | 4.577718 | -5.093474 | 1.373185 |
| H | 4.476125 | -5.228414 | -0.388980 |
| N | -5.839151 | -3.501336 | 0.081753 |
| C | -5.270149 | -4.833124 | 0.146102 |
| C | -7.283017 | -3.351149 | 0.038430 |
| H | -6.071238 | -5.565483 | 0.137408 |
| H | -4.618150 | -5.030919 | -0.708650 |
| H | -4.686557 | -4.976081 | 1.059419 |
| H | -7.740777 | -4.334958 | 0.067141 |
| H | -7.656057 | -2.781738 | 0.892843 |
| H | -7.611089 | -2.854192 | -0.877532 |
| S | -0.136792 | -0.157510 | 3.121616 |
| H | -1.431538 | 0.209836 | 3.138275 |

**$S_1$**

| | | | |
|---|---|---|---|
| N | 1.363027 | 1.143142 | 0.022220 |
| N | -1.329911 | 1.226142 | 0.001779 |
| O | 1.443344 | -1.690894 | 0.382541 |
| O | -1.468833 | -1.588670 | 0.010817 |
| C | 5.541924 | -1.335841 | 0.103079 |
| C | 2.716566 | -1.547062 | 0.293573 |
| C | 4.768165 | -0.222027 | 0.028417 |
| C | 4.932232 | -2.620862 | 0.270217 |
| C | 3.541130 | -2.680426 | 0.360465 |
| C | 3.350834 | -0.249337 | 0.111736 |
| C | 0.767540 | 2.332967 | -0.133262 |
| C | -0.667514 | 2.378875 | -0.130700 |
| C | -1.341089 | 3.613885 | -0.290012 |
| N | -1.925505 | 4.599452 | -0.420171 |
| C | -2.742430 | -1.416495 | 0.043709 |
| C | -5.567827 | -1.131624 | 0.077525 |

| | | | |
|---|---|---|---|
| C | -3.354652 | -0.094220 | 0.051497 |
| C | -3.589793 | -2.534092 | 0.053949 |
| C | -4.982098 | -2.438362 | 0.067811 |
| C | -4.772305 | -0.030209 | 0.063909 |
| C | -2.650125 | 1.117868 | -0.009513 |
| H | -3.249154 | 2.025526 | -0.087220 |
| C | 2.676712 | 0.973464 | -0.023672 |
| H | 3.304240 | 1.848445 | -0.193285 |
| H | 3.029912 | -3.621386 | 0.492306 |
| H | 6.613674 | -1.241272 | 0.030006 |
| H | 5.249311 | 0.740101 | -0.104896 |
| H | -3.096446 | -3.493588 | 0.047134 |
| H | -6.639341 | -1.010156 | 0.088903 |
| H | -5.235651 | 0.949733 | 0.065342 |
| C | 1.512148 | 3.522170 | -0.317036 |
| N | 2.148205 | 4.472929 | -0.464209 |
| Zn | -0.028236 | -0.373800 | 0.712930 |
| N | 5.701042 | -3.737162 | 0.336305 |
| C | 7.148684 | -3.656412 | 0.243115 |
| C | 5.076337 | -5.036795 | 0.487161 |
| H | 7.563606 | -4.655215 | 0.332202 |
| H | 7.469227 | -3.241863 | -0.715550 |
| H | 7.569812 | -3.044739 | 1.043873 |
| H | 5.844175 | -5.803482 | 0.507621 |
| H | 4.504824 | -5.099083 | 1.416839 |
| H | 4.399568 | -5.251708 | -0.343934 |
| N | -5.773760 | -3.540720 | 0.073909 |
| C | -5.174687 | -4.860869 | 0.059867 |
| C | -7.221675 | -3.423225 | 0.091523 |
| H | -5.959417 | -5.610478 | 0.063058 |
| H | -4.561504 | -5.010466 | -0.832639 |
| H | -4.543456 | -5.021590 | 0.937703 |
| H | -7.655535 | -4.417978 | 0.095713 |
| H | -7.572544 | -2.898676 | 0.983095 |
| H | -7.594229 | -2.896538 | -0.790016 |
| S | -0.206211 | -0.104336 | 3.034088 |
| H | -1.358070 | 0.590007 | 2.992608 |

Complex **3 + 2HS$^{-}$**

**$S_0$**

| | | | |
|---|---|---|---|
| N | 1.334184 | 1.144529 | 0.166485 |
| N | -1.327967 | 1.182014 | 0.054245 |
| O | 1.442798 | -1.666360 | 0.555200 |
| O | -1.522467 | -1.623259 | 0.350878 |
| C | 5.524203 | -1.270120 | 0.063421 |
| C | 2.708159 | -1.501401 | 0.399849 |
| C | 4.733309 | -0.167315 | 0.031485 |
| C | 4.931355 | -2.557484 | 0.255934 |
| C | 3.548923 | -2.631147 | 0.416408 |
| C | 3.325721 | -0.212559 | 0.191213 |

| | | | |
|---|---|---|---|
| C | 0.710343 | 2.345865 | -0.057544 |
| C | -0.656064 | 2.365265 | -0.113466 |
| C | -1.350213 | 3.591267 | -0.356566 |
| N | -1.944303 | 4.554861 | -0.549104 |
| C | -2.782510 | -1.418251 | 0.206383 |
| C | -5.592026 | -1.092768 | -0.113338 |
| C | -3.361639 | -0.106395 | 0.026800 |
| C | -3.658791 | -2.521539 | 0.206213 |
| C | -5.038848 | -2.401511 | 0.054784 |
| C | -4.767471 | -0.014578 | -0.129481 |
| C | -2.622513 | 1.083385 | -0.072791 |
| H | -3.202490 | 1.984495 | -0.287590 |
| C | 2.625715 | 0.997802 | 0.059979 |
| H | 3.237583 | 1.873318 | -0.170727 |
| H | 3.054012 | -3.578528 | 0.562695 |
| H | 6.589015 | -1.166257 | -0.069089 |
| H | 5.194706 | 0.800871 | -0.129291 |
| H | -3.193979 | -3.486399 | 0.337018 |
| H | -6.653801 | -0.953770 | -0.238149 |
| H | -5.199159 | 0.970337 | -0.269937 |
| C | 1.456018 | 3.549179 | -0.255027 |
| N | 2.089094 | 4.495054 | -0.407181 |
| Zn | -0.045853 | -0.356488 | 0.874038 |
| N | 5.710404 | -3.671127 | 0.278881 |
| C | 7.144753 | -3.581122 | 0.071028 |
| C | 5.099473 | -4.978553 | 0.416168 |
| H | 7.575955 | -4.573935 | 0.153071 |
| H | 7.389689 | -3.186298 | -0.918514 |
| H | 7.620378 | -2.947797 | 0.822514 |
| H | 5.874541 | -5.738574 | 0.408291 |
| H | 4.548840 | -5.064566 | 1.356055 |
| H | 4.406908 | -5.187009 | -0.404206 |
| N | -5.852646 | -3.490023 | 0.064523 |
| C | -5.285511 | -4.816646 | 0.204799 |
| C | -7.288259 | -3.349355 | -0.102013 |
| H | -6.083882 | -5.551747 | 0.180621 |
| H | -4.588587 | -5.042443 | -0.606809 |
| H | -4.750834 | -4.925244 | 1.151968 |
| H | -7.747610 | -4.331234 | -0.045264 |
| H | -7.726311 | -2.728835 | 0.682817 |
| H | -7.544301 | -2.912721 | -1.070568 |
| S | -0.200122 | -0.004204 | 3.210056 |
| H | -1.533405 | 0.180137 | 3.215689 |
| S | 0.578068 | -0.877019 | -3.249756 |
| H | 0.854443 | -1.044416 | -1.942088 |

| | $\Phi_F$ | $A_{max}$ | $F_{max}$ |
|---|---|---|---|
| Complex **1** | 0.01 | $A_{370}$ = 0,216 | $F_{662}$ = 9,4 |
| Complex **1_HS** | 0.17 | $A_{378}$ = 0,449 | $F_{671}$ = 170 |
| Complex **2** | 0.07 | $A_{412}$ = 0,08 | $F_{491}$ = 10 |
| Complex **2_HS** | 0.13 | $A_{366}$ = 0,285 | $F_{491}$ = 109 |
| Complex **3** | 0.4 | $A_{581}$ = 1,1 | $F_{646}$ = 470 |
| Complex **3_HS** | 0.08 | $A_{436}$ = 0,761 | $F_{637}$ = 14,5 |
| Complex **4** | 0.2 | $A_{570}$ = 0,353 | $F_{627}$ = 200 |
| Complex **4_HS** | 0.6 | $A_{596}$ = 0,485 | $F_{635}$ = 803 |

**Table S2.** Photophysical features of complexes **1-4**.
